# AKF-D52, a Synthetic Phenoxypyrimidine-Urea Derivative, Triggers Extrinsic/Intrinsic Apoptosis and Cytoprotective Autophagy in Human Non-Small Cell Lung Cancer Cells

**DOI:** 10.3390/cancers13225849

**Published:** 2021-11-22

**Authors:** Hyo-Sun Gil, Jeong-Hun Lee, Ahmed K. Farag, Ahmed H. E. Hassan, Kyung-Sook Chung, Jung-Hye Choi, Eun-Joo Roh, Kyung-Tae Lee

**Affiliations:** 1 Department of Pharmaceutical Biochemistry, College of Pharmacy, Kyung Hee University, 26, Kyungheedae-ro, Seoul 02447, Korea; kilhs5654@khu.ac.kr (H.-S.G.); ztztzt08@khu.ac.kr (J.-H.L.); adella76@khu.ac.kr (K.-S.C.); 2 Department of Life and Nanopharmaceutical Sciences, Graduate School, Kyung Hee University, 26, Kyungheedae-ro, Seoul 02447, Korea; jchoi@khu.ac.kr; 3 Manufacturing Department, Curachem, Inc., Cheongju-si 28161, Chungcheongbuk-do, Korea; ahmed@curachem.com; 4 Department of Medicinal Chemistry, Faculty of Pharmacy, Mansoura University, Mansoura 35516, Egypt; ahmed_hassan@mans.edu.eg; 5 Oriental Pharmaceutical Science, College of Pharmacy, Kyung Hee University, 26, Kyungheedae-ro, Seoul 02447, Korea; 6 Department of Biomedical and Pharmaceutical Sciences, Graduate School, Kyung Hee University, 26, Kyungheedae-ro, Seoul 02447, Korea; 7 Division of Bio-Medical Science &Technology, KIST School, University of Science and Technology, Seoul 02792, Korea; r8636@kist.re.kr

**Keywords:** AKF-D52, non-small cell lung cancer (NSCLC), apoptosis, autophagy, reactive oxygen species (ROS)

## Abstract

**Simple Summary:**

We previously reported the antiproliferative effects of a phenoxypyridine urea derivative. In this study, we aimed to investigate the antiproliferative effects of 1-(3,5-dimethoxyphenyl)-3-(4-(3-methoxyphenoxy)-2-((4-morpholinophenyl)amino)pyrimidin-5-yl)urea (AKF-D52) in non-small cell lung cancer cells. We found that (i) AKF-D52 induces apoptosis in caspase-dependent and caspase-independent pathways; (ii) AKF-D52-induced apoptosis is caused by the clustering of a death-inducing signaling complex and mitochondrial-dependent signaling; (iii) AKF-D52 induces cytoprotective autophagy, and pre-treatment with an autophagy inhibitor enhances the apoptotic effect of AKF-D52; and (iv) AKF-D52-induced apoptosis and autophagy are attenuated by the reactive oxygen species (ROS) scavenger α-tocopherol. Furthermore, AKF-D52 suppressed tumor growth in a xenograft mouse model. Collectively, our findings regarding the efficacy and molecular mechanisms of AKF-D52 identify this compound as a potential therapeutic agent for the treatment of lung cancer.

**Abstract:**

Previously, we discovered that 1-(3,5-dimethoxyphenyl)-3-(4-(3-methoxyphenoxy)-2-((4-morpholinophenyl)amino)pyrimidin-5-yl)urea (AKF-D52), a synthetic phenoxypyrimidine urea derivative, acts as a growth inhibitor of various cancer cell types. In this study, we elucidated the antiproliferative properties of AFK-D52 and underlying mechanisms in non-small cell lung cancer (NSCLC) cells and an A549 xenograft animal model. AKF-D52 was found to induce both caspase-dependent and -independent apoptotic cell death. Furthermore, the mitochondrial component of the AKF-D52-induced apoptosis mechanism involves a reduction in mitochondrial membrane potential and regulation in B cell lymphoma-2 family protein expression. Moreover, AKF-D52 activates the extrinsic pathway through up-regulated expression of death receptor 3 and Fas and then the formation of a death-inducing signaling complex. AKF-D52 also induced autophagy by increasing acidic vesicular organelle formation and microtubule-associated protein 1A/1B-light chain 3-II levels and reducing p62 levels. Notably, pretreatment with autophagy inhibitors enhanced AKF-D52-induced cell death, indicating that the induced autophagy is cytoprotective. AKF-D52 treatment also triggered reactive oxygen species (ROS) production in NSCLC cells, whereas the antioxidant α-tocopherol abolished AKF-D52-induced cell death. In a xenograft lung cancer mouse model, AKF-D52 administration attenuated tumor growth by inducing apoptosis and autophagy in tumor tissues. Collectively, our data indicate that AKF-D52-induced ROS production plays a role in mediating apoptosis and cytoprotective autophagy in NSCLC.

## 1. Introduction

Lung cancer is associated with a poor prognosis, typically with a low 5-year survival rate (15%), and is the most severe type of malignant cancer [1]. Pathological classification distinguishes lung cancers as either non-small cell lung cancer (NSCLC, 85%) or small cell lung cancer (SCLC, 15%), each type of which requires a distinct therapeutic approach [2]. Therapeutic protocols for NSCLC include surgery, chemotherapy, targeted therapy, and radiation therapy [3]. However, more effective treatments with reduced side effects and drug resistance are desirable [4].

Dysregulated apoptosis plays a pivotal pathological role in numerous diseases, including cancer. This cellular process entails programmed cell death mediated via caspase activation and is characterized by morphological changes, including chromatin condensation, nuclear fragmentation, and cell shrinkage [5]. Apoptosis may arise from one of two different pathways: an extrinsic signaling pathway (death receptor pathway) initiated by transmembrane receptor-mediated interactions or an intrinsic signaling pathway (mitochondrial pathway) induced by non-receptor-mediated stimuli, such as radiation, toxins, hypoxia, or free radicals [6]. Given that apoptosis dysregulation generally occurs within cancer cells, the induction of apoptosis is considered an effective strategy for cancer therapy [7].

Autophagy is a self-degradation process in which double-membrane autophagosomes isolate cellular components and combine with lysosomes to initiate degradation by resident hydrolytic enzymes [8]. Autophagy plays a role in both the regression and development of tumor growth, and therefore, an appropriate level of autophagic regulation is required for cancer cells [9]. Numerous studies have established that autophagic cell death [10,11,12] or cytoprotective autophagy [13,14] can occur in cancer cells, and consequently, compounds that regulate autophagy in cancer might serve as chemotherapeutic agents or adjunctive treatments that could be used as alternatives to conventional anticancer drugs.

Reactive oxygen species (ROS) are generated by oxygen consumption during normal cellular metabolism [15]. Within cancer cells, moderate levels of ROS can promote a diverse range of cellular responses, including survival, angiogenesis, and metastasis; however, excessive ROS production can cause apoptotic cell death [16]. Several cytotoxic agents have been shown to induce ROS-induced apoptosis and autophagy in different cancer types [17,18,19]. Accordingly, ROS formation is associated with chemotherapy or radiation therapy by influencing downstream cancer cell survival or death signaling pathways [20].

To date, a range of diarylureas that bind to certain biomolecules has been designed and developed [21]. Among these, sorafenib is a well-established diarylurea-derived anticancer agent that has been approved by the Food and Drug Administration for the treatment of hepatocellular and renal carcinomas [22], and several other diarylurea-based compounds, including regorafenib, have been found to possess antitumor effects [23,24,25].

In a previous study, we found that a novel phenoxypyrimidine urea derivative, 1-(3,5-dimethoxyphenyl)-3-(4-(3-methoxyphenoxy)-2-((4-morpholinophenyl)amino)pyrimidin-5-yl)urea (AKF-D52), a diarylurea-2,4-disubstituted pyrimidine hybrid compound, showed antiproliferative properties in various cancer cells and inhibitory effect on multiple kinases, especially inhibition against Feline McDonough Sarcoma (FMS) and c-KIT. However, as the inhibition of the AKF-D52 on these protein tyrosine kinases is lower relative to sorafenib and pazopanib, it might only help to explain the evoked antiproliferative activity partially [26]. Notably, AKF-D52 induced apoptotic cell death in A549 lung cancer cells, but the molecular mechanisms underlying the growth inhibitory efficacy of AKF-D52 in human NSCLC cells have yet to be elucidated. In the current study, we investigated the therapeutic potential of AKF-D52 in human NSCLC cells based on both in vitro and in vivo experiments and characterized the molecular mechanisms underlying its ameliorative effects.

## 2. Materials and Methods

### 2.1. Reagents and Chemicals

**Figure 1:**
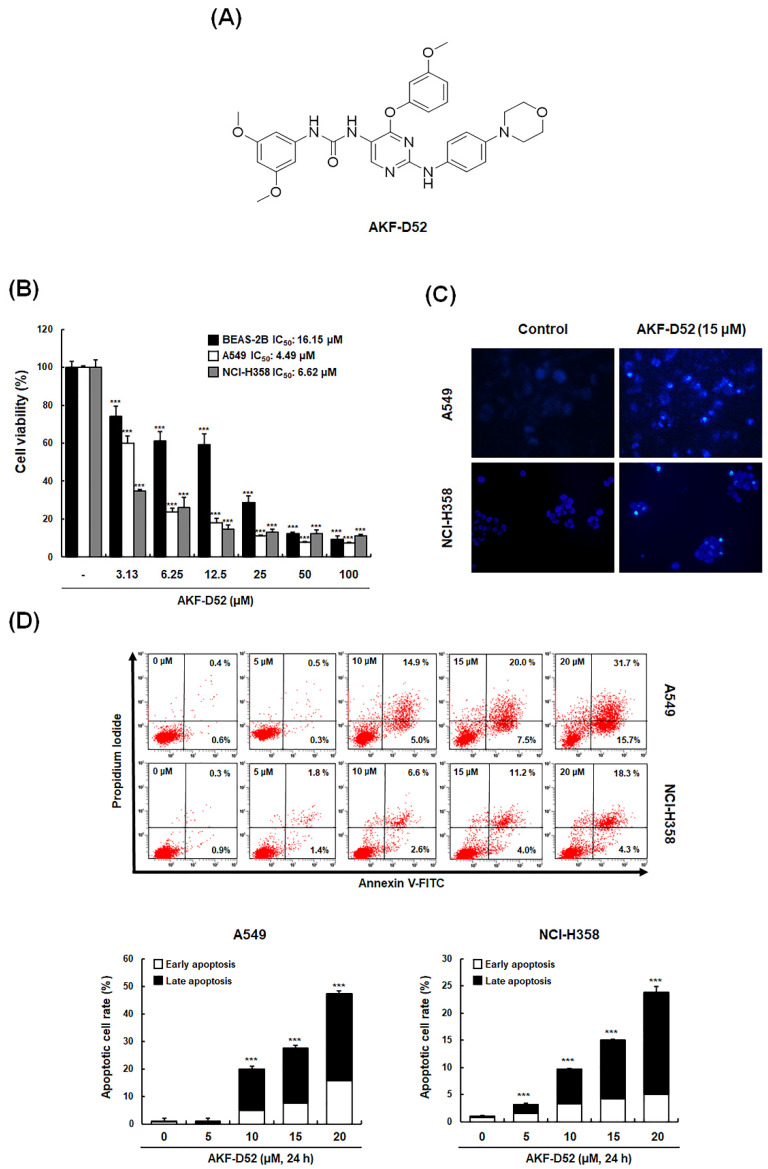
The effects of AKF-D52 on NSCLC cell viability. (**A**) The structure of AKF-D52. (**B**) A549, NCI-H358, and BEAS-2B cells were treated with various concentrations (3.12–100 μM) of AKF-D52 for 48 h, and cell viability was measured using the MTT assay. (**C**) AKF-treated cells were stained with DAPI solution for detection of chromatin condensation. (**D**) AKF-treated cells were co-stained with PI and FITC-conjugated annexin V, and stained cells were by flow cytometry. Data are presented as the mean ± SD of three independent experiments, and significant differences are denoted as *** p < 0.001 vs. the control group.

AKF-D52 (Figure 1A) was prepared as previously described [26]. Fetal bovine serum (FBS), penicillin/streptomycin (PS), Minimum Essential Medium (MEM), and RPMI 1640 medium were obtained from Life Technologies Inc (Chicago, IL, USA). Acridine orange, carbonyl cyanide m-chlorophenylhydrazone (CCCP), chloroquine (CQ), 4′,6-diamidino-2-phenylindole (DAPI), 3,3′-dihexyloxacarbocyanine iodide (DiOC_6_), dimethyl sulfoxide (DMSO), 2′,7′-dichlorodihydrofluorescein diacetate (H_2_DCFDA), 3-(4,5-dimethylthiazol-2-yl)-2,5-diphenyltetrazolium bromide (MTT), Nonidet P-40 (NP-40), propidium iodide (PI), phenylmethylsulfonyl fluoride (PMSF), protein inhibitor cocktail, ribonuclease A (RNase A), N,N,N′,N′-tetramethylethylenediamine (TEMED), and α-tocopherol were purchased from Sigma Aldrich (St. Louis, MO, USA). Antibody for caspase-8 (CAT#551242) and FITC (fluorescein isothiocyanate)-Annexin V Apoptosis Detection Kit I was purchased from BD Bioscience pharmigen (San Jose, CA, USA). carbobenzoxy-valyl-alanyl-aspartyl-[O-methyl]-fluoromethylketone (z-VAD-fmk) was purchased from R&D systems (Minneapolis, MN, USA). Antibodies for apoptosis-inducing factor (AIF) (sc-9417), Bad (sc-8044), B cell lymphoma-2 (Bcl-2) (sc-7382), Bid (sc-6539), Bim (sc-374358), caspase-3 (sc-271759), death receptor 3 (DR3) (sc-7909), endonuclease G (Endo G) (sc-365359), Fas (sc-8009), Fas-associated protein with death domain (FADD) (sc-271748), Fas ligand (FasL) (sc-6237), IκBα (sc-371), Noxa (sc-56169), nucleolin (sc-55486), poly (ADP-ribose) polymerase (PARP)-1 (sc-8009), p62 (sc-28359), p65 (sc-8008), tumor necrosis factor-like weak inducer of apoptosis (TWEAK) (sc-56248), α-tublin (sc-5286), and β-actin (sc-81178) were purchased from Santa Cruz Biotechnology (Santa Cruz, CA, USA). Antibodies for AMP-activated protein kinase (AMPK) (#2532), Akt (#9272), caspase-9 (#9502), cleaved caspase-3 (#9661), cytochrome *c* oxidase (COX) IV (#4850), cytochrome *c* (#4272), Smac/DIABLO (#15108), microtubule-associated protein 1A/1B-light chain 3 (LC3) (#4108), mammalian target of rapamycin (mTOR) (#2972), phospho-AMPK (#2535), phospho-Akt (#9271), phospho-IκBα (#2859), phospho-mTOR (#2971), and phospho-p65 (#3031) were purchased from Cell Signaling Technology (Danvers, MA, USA). All chemicals and reagents above were diluted to working concentrations before use.

### 2.2. Cell Culture

NSCLC cells (A549 and NCI-H358) and normal lung epithelial cells (BEAS-2B) were obtained from the Korean Cell Line Bank. A549 and NCI-H358 cells were cultured in RPMI 1640 medium, and BEAS-2B cells were cultured in MEM supplemented with 10% inactivated FBS and 1% PS (100 U/mL). Cells were then maintained at 37 °C in an atmosphere of 5% CO_2_ in the air. Cell lines were routinely checked for mycoplasma contamination by e-Myco™ plus Mycoplasma PCR Detection Kit (Intron Biotechnology, Seongnam, Republic of Korea).

### 2.3. MTT Assay

The cell viability was measured by MTT assay, as previously described [27].

### 2.4. DAPI Staining

To detect the amount of fragmented DNA in apoptotic cells, cells were treated with AKF-D52 (20 μM) for 24 h. Then cells were washed three times with ice-cold PBS and fixed with 4% paraformaldehyde for 30 min. The DAPI staining solution was added to the cells. After incubation in the dark for 30 min at room temperature, the apoptotic cells were observed under a fluorescence microscope (200× magnification).

### 2.5. Annexin V/PI Staining for Apoptosis Analysis

To detect AKF-D52-induced apoptotic cells, the cells were treated with AKF-D52 (0–20 μM) for 24 h. Then cells were harvested and suspended with 100 μL of binding buffer (10 mM HEPES/NaOH, 140 mM NaCl, 2.5 mM CaCl_2_, pH 7.4) and stained with 5 μL of FITC-conjugated Annexin V and 5 μL of PI (50 µg/mL). After incubation in the dark for 15 min at room temperature, the cells were analyzed by flow cytometric analysis (Beckman Coulter, CA, USA).

### 2.6. Nuclear Extraction

After treatment of cells with AKF-D52 (0–20 μM) for 24 h, AKF-D52-treated cells were harvested and washed with PBS and pelleted by centrifugation. Cell pellets were resuspended in hypotonic buffer (10 mM HEPES (pH 7.9), 10 mM KCl, 0.1 mM ethylenediaminetetraacetic acid (EDTA), 0.1 mM ethylene glycol bis(2-aminoethyl ether)-N,N,N′,N′-tetraacetic acid (EGTA), 0.5 mM PMSF, 1 mM DTT, and protease inhibitor cocktail (PIC) and incubated on ice for 15 min. Cells were then lysed by adding 0.1% NP-40 and vortexed vigorously for 10 s. Nuclei were pelleted by centrifugation at 12,000× *g* for 1 min at 4 °C and resuspended in high salt buffer (20 mM HEPES, pH 7.9, 25% glycerol, 400 mM KCl, 1.5 mM MgCl2, 0.2 mM EDTA, 0.5 mM DTT, 1 mM NaF, 1 mM sodium orthovanadate).

### 2.7. Western Blot Analysis

AKF-D52-treated cells were pelleted by centrifugation (2500 rpm, 10 min, 4 °C). The cell pellet was lysed using protein lysis buffer (Intron, Seoul, Republic of Korea). Total protein concentrations were measured by the Bradford assay. After heat denaturation for 5 min, proteins were resolved by 8–15% SDS-PAGE gels and transferred to polyvinylidene difluoride (PVDF) membranes. The membranes were blocked in 5% skim milk for 1 h at room temperature and then incubated with specific primary antibodies. After three times washing with Tween 20/Tris-buffered saline (T/TBS), the membrane was incubated with horseradish peroxidase-conjugated secondary antibody for 2 h at room temperature. Following three washes in T/TBS, Specific bands were visualized through Image Quant LAS-4000 (Fujifilm Life Science, Tokyo, Japan) using an ECL chemiluminescence substrate (Santa Cruz Biotechnology, Santa Cruz, CA, USA). All original western blot figures are included in Figures S2–S14.

### 2.8. Analysis of Mitochondrial Membrane Potential (ΔΨm)

After treatment of cells with AKF-D52 (0–20 μM) for 24 h, AKF-D52-treated cells were incubated with 30 nM DiOC_6_ for 30 min in the dark. Cells were harvested and washed with PBS and pelleted by centrifugation. Fluorescence was measured using flow cytometric analysis (Beckman Coulter, CA, USA).

### 2.9. Preparation of Cytosolic and Mitochondrial Fractionation

Mitochondrial and cytosolic fraction was obtained according to the method previously described [27].

### 2.10. Immunoprecipitation Assay

Immunoprecipitation assay was examined according to the method previously described [28].

### 2.11. Acridine Orange Staining

After cells were seeded in a 4-well plate for 24 h, the cells were treated with AKF-D52 and stained with 1 μg/mL acridine orange at 37 °C in the dark for 15 min. Then, the cells were fixed with 4% formaldehyde for 10 min and washed in T/TBS before DAPI staining. Subsequently, the cells were washed by PBS and observed under a fluorescence microscope.

### 2.12. Measurement of Reactive Oxygen Species (ROS)

To measure the intracellular ROS level, cells were treated with AKF-D52 (20 μM) for 2 h. then cells were washed with PBS and stained with H_2_DCFDA in the dark for 30 min. After a three-time washing step, cells were analyzed by flow cytometry.

### 2.13. Animals

The male BALB/c nude mice (6-week-old, 20–23 g) were purchased from Nara Biotec Co. (Pyeongtaek, Republic of Korea). All experiment processes were carried out under university guidelines as described previously [27] and were approved by the ethical committee for Animal Care and Use of Kyung Hee University according to the animal protocol (KHUASP(SE)-20-191).

### 2.14. In Vivo Tumor Xenograft Studies

After 7 days of acclimation, A549 cells were subcutaneously injected into male BALB/c nude mice. The tumor size of each animal was measured using calipers and calculated as V = π/6 × (length) × (width)^2^ [29]. After tumor volume reached about 250 mm^3^, mice were divided into 4 groups (*n* = 6) and treated with vehicle (DMSO:Cremophor:D.W. = 1:1:8, intraperitoneal (i.p.)), paclitaxel (positive control, 5 mg/kg, i.p.) and AKF-D52 (10 or 30 mg/kg, i.p.). During the treatment, tumor volume and body weight were measured twice per week for 4 weeks. On day 30, mice were sacrificed, and tumors were obtained.

### 2.15. Measurement of Alanine Aminotransferase (ALT), Aspartate Aminotransferase (AST), and Blood Urea Nitrogen (BUN) Levels

The plasma from mice in each group was obtained, and ALT, AST, and BUN levels in the plasma were measured by using kits from Gy (Gwangju, Gyeonggi Province, Republic of Korea).

### 2.16. Statistical Analysis

Data were analyzed and visualized using GraphPad Prism^®^ Version 8.0.1 software (GraphPad Software Inc., La Jolla, CA, USA). Data are presented as the mean ± SD of triplicate experiments. Statistical significance was identified by unpaired *t*-test, and *p-*values of <0.05 were considered statistically significant.

## 3. Results

### 3.1. AKF-D52 Suppresses Cell Viability via Apoptotic Cell Death in A549 and NCI-H358 Cells

To investigate the effects of AKF-D52 on cell viability, A549, NCI-H358, and normal lung epithelial cells (BEAS-2B) cells were treated with different concentrations of AKF-D52 (3.13–100 μM) to determine IC_50_ values. As shown in Figure 1B, AKF-D52 suppressed the cell viability in a concentration-dependent manner, with IC_50_ values of 4.49 μM and 6.62 μM against A549 and NCI-H358, respectively, whereas it showed a higher IC_50_ value (16.15 μM) in BEAS-2B cells. By statistical analysis, we found that the IC_50_ value of BEAS-2B showed significance compared with that of A549 (*p*-value: 0.0002) or NCI-H358 (*p*-value: 0.0007), indicating the selective cytotoxicity of AKF-D52 against NSCLC cells rather than normal cells. To assess whether the observed reduction in A549 and NCI-H358 cell viability induced by AFK-D52 was mediated via the induction of apoptosis, we examined changes in cell nucleus morphology and the externalization of phosphatidylserine using DAPI staining and an Annexin V-FITC/PI assay, respectively. As shown in Figure 1C,D, AKF-D52-treated cells were characterized by condensed nuclei and a significant dose-dependent increase in Annexin V/PI-positive cells. These results accordingly indicated that AKF-D52 reduced the viability of A549 and NCI-H358 NSCLC cells by inducing apoptotic cell death.

### 3.2. AKF-D52 Concurrently Induces Caspase-Dependent and -Independent Apoptosis in A549 and NCI-H358 Cells

**Figure 2:**
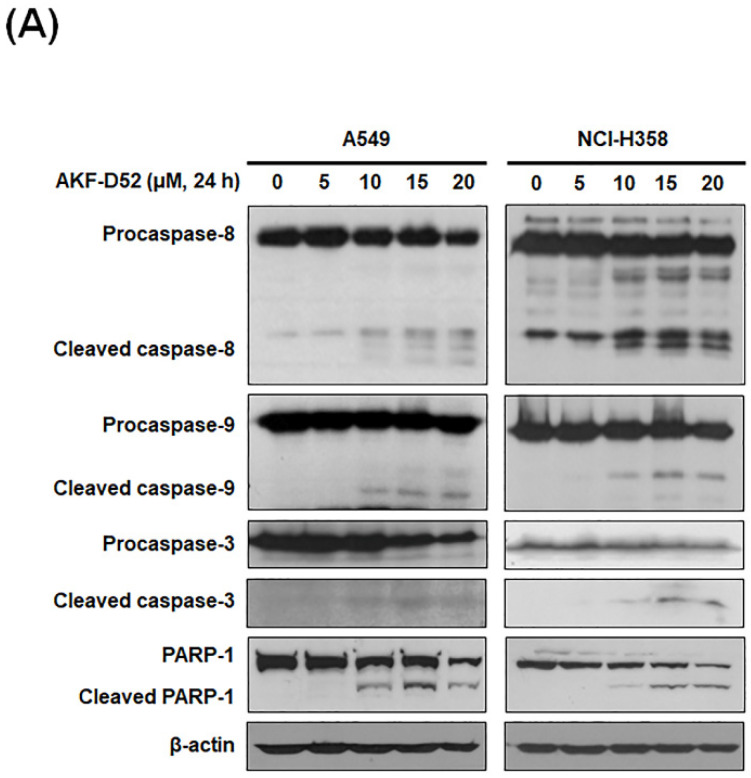

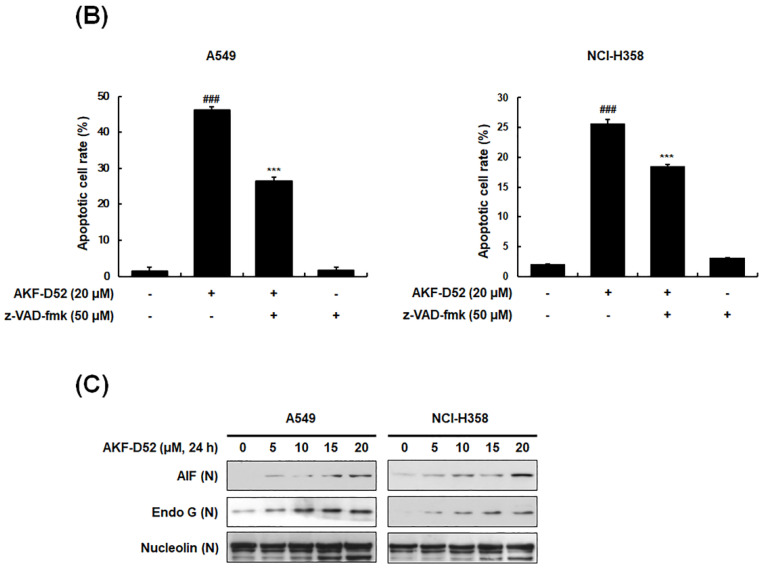
The effects of AKF-D52-induced apoptosis on caspase-dependent and -independent pathways in NSCLC cells. Cells were treated with different concentrations (0, 5, 10, 15, or 20 μM) of AKF-D52 for 24 h. (**A**) Cell lysates were prepared, and Western blot analysis was performed as described in the *Materials and Methods* section. β-actin was used as an internal control. (**B**) After pretreatment with 50 µM z-VAD-fmk, cells were treated with AKF-D52 (20 μM) for 24 h, and the apoptotic cell rate was analyzed based on PI staining using flow cytometry. (**C**) Cells were harvested, and nuclear fraction was obtained as described in the *Materials and Methods* section. Nucleolin was used as an internal control. Data are presented as the mean ± SD of three independent experiments, and significant differences are denoted as *^###^ p* < 0.001 vs. the untreated control group, *** p < 0.001 vs. the AKF-D52-treated group.

The regulation of apoptosis is primarily dependent on the activation of caspases [30]. Accordingly, we examined the cleavage of caspases (caspase-8, -9, and -3 and PARP-1, which are hallmarks of caspase-dependent apoptosis. As shown in Figure 2A, AKF-D52 promoted the cleavage of caspase-8, -9, and -3, and PARP-1 in a concentration-dependent manner. Interestingly, pretreatment with z-VAD-fmk (a broad caspase inhibitor) only partially blocked AKF-D52-induced apoptosis in NSCLC cells (Figure 2B), thereby indicating that AKF-D52-induced apoptosis is only partially caspase-dependent. Accordingly, we subsequently evaluated the effects of AKF-D52 on caspase-independent pathways. As shown in Figure 2C, AKF-D52 induced the nuclear translocation of AIF and Endo G in A549 and NCI-H358 cells. These findings thus indicate that AKF-D52-induced apoptosis in NSCLC cells involves both caspase-dependent and -independent pathways.

### 3.3. AKF-D52-Induced Apoptosis Is Dependent on Mitochondrial Dysfunction in A549 and NCI-H358 Cells

**Figure 3:**
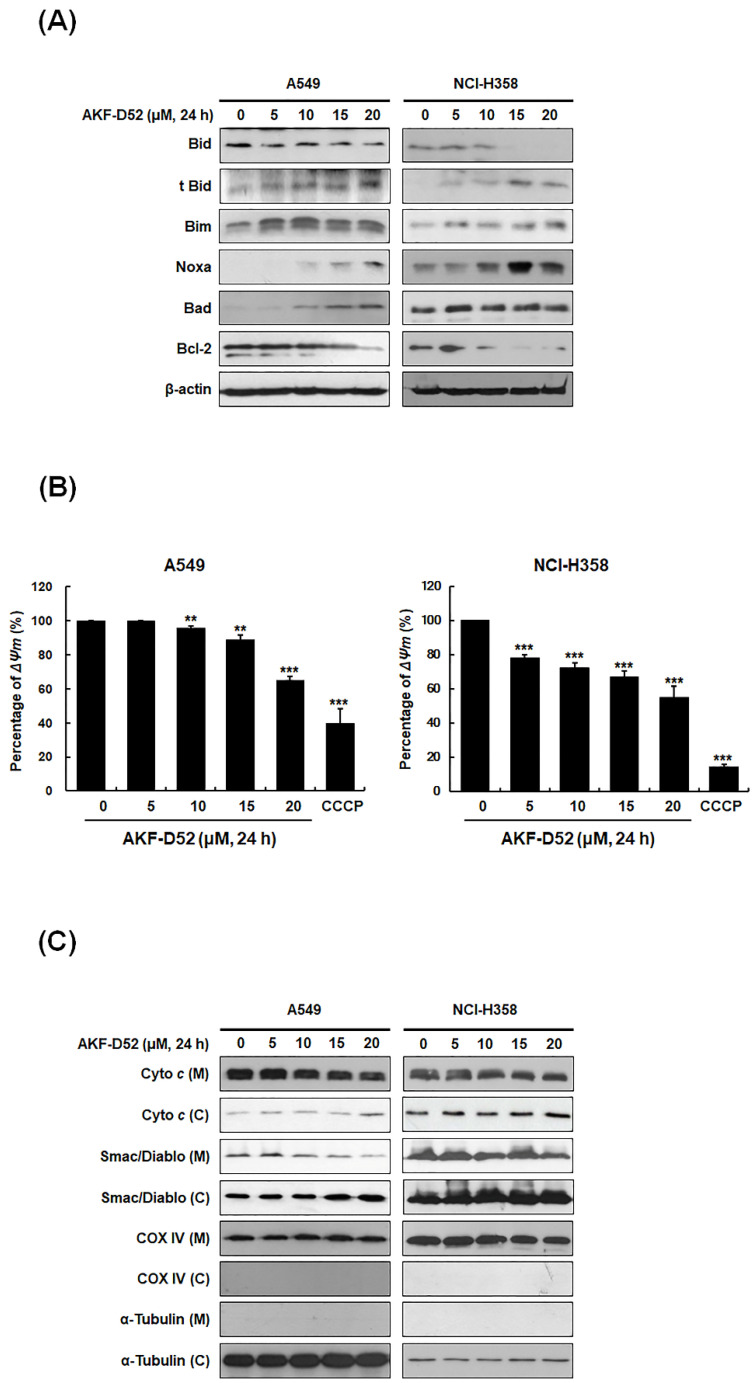
The effects of AKF-D52 on activation of the intrinsic pathway in NSCLC cells. Cells were exposed to different concentrations of AKF-D52 (0, 5, 10, 15, or 20 μM) for 24 h. (**A**) Cell lysates were prepared, and cellular proteins were resolved by SDA-PAGE to determine the expression of Bid, t-Bid, Bim, Noxa, Bad, and Bcl-2. β-actin was used as an internal control. (**B**) Cells were stained with DiOC_6_, and *ΔΨm* was analyzed by flow cytometry. (**C**) Cells were harvested and mitochondrial or cytosolic fractions were obtained, as described in the *Materials and Methods* section. COX IV and α-tubulin were used as internal controls. Data are presented as the mean ± SD of three independent experiments, and significant differences are denoted as ** p < 0.01, *** p < 0.001 vs. the untreated control group.

Given our observations that AKF-D52 triggers both caspase-dependent and caspase-independent apoptosis, we postulated that the apoptosis-inducing effects of AKF-D52 include a mitochondrial pathway involving outer membrane permeabilization by Bcl-2 family proteins. To test this hypothesis, we examined the expression of Bcl-2 family proteins in A549 and NCI-H358 cells. AKF-D52 was accordingly found to upregulate the expression of BH3-only proteins (Bim, Noxa, and Bad) and a truncated form of Bid (t-Bid), whereas the levels of Bcl-2 and Bid expression were reduced in NSLCLC cells (Figure 3A). As Bcl-2 family proteins trigger the disruption of mitochondrial membrane potential (*ΔΨm*), we further examined the effects of AKF-D52 on the changes in *ΔΨm* using DiOC_6_. As shown in Figure 3B, treatment of A549 and NCI-358 cells with AKF-D52 reduced *ΔΨm* in a concentration-dependent manner. In addition, AKF-D52 was observed to induce the translocation of cytochrome *c* and Smac/DIABLO from mitochondria to the cytosol, indicating that the disruption of *ΔΨm* leads to the cytosolic release of apoptosis-inducing mitochondrial proteins in NSCLC cells (Figure 3C). Taken together, these findings indicate that AKF-D52-induced apoptosis is dependent on mitochondrial dysfunction and thus the involvement of the intrinsic pathway.

### 3.4. AKF-D52-Induced Apoptosis Requires Death-Inducing Signaling Complex Activation in A549 and NCI-H358 Cells

**Figure 4:**
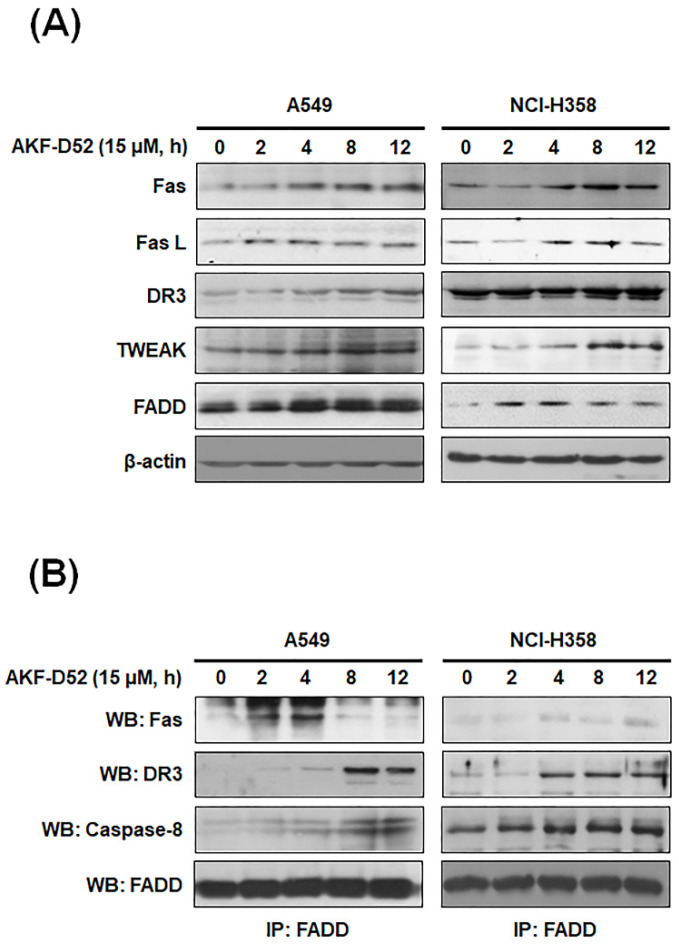

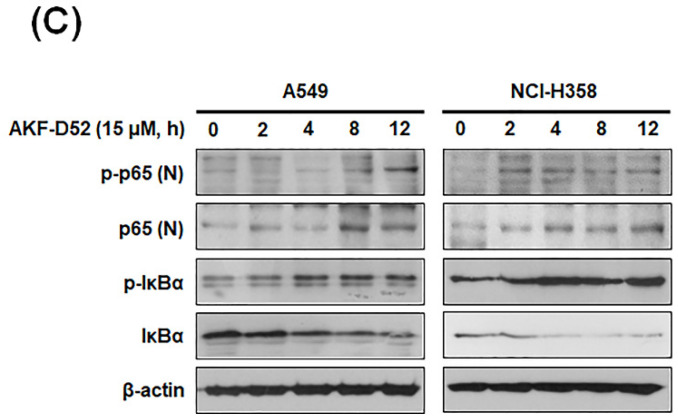
The effects of AKF-D52 on activation of the extrinsic pathway in NSCLC cells. A549 and NCI-H358 cells were treated with 15 µM AKF-D52 for different lengths of time (0, 2, 4, 8, or 12 h). (**A**) Cells were lysed, and Western blot analysis was performed, as described in the *Materials and Methods* section. β-actin was used as an internal control. (**B**) Cell lysates were prepared, and an immunoprecipitation assay was performed, as described in the *Materials and Methods* section. FADD was used as an internal control. (**C**) Cells were lysed, and nuclear (N) or cellular proteins were separated by SDS-PAGE to determine the expression of phospho-p65 (N), p65 (N), p-IκBα, and IκBα. β-actin was used as an internal control.

The extrinsic pathway of apoptosis involves the formation of a death-inducing signaling complex (DISC) via the clustering of death ligands, receptors, and domains [31]. This complex activates caspase-8 and cleaves Bid, which, in turn, disrupts mitochondrial function and promotes the activation of caspase-9 in the intrinsic pathway [32]. In this regard, we investigated the expression of DISC constituent proteins in A549 and NCI-H358 cells. AKF-D52 increases the expression of Fas, FasL, DR3, TWEAK, and FADD (Figure 4A). In addition, we examined the association of Fas, DR3, or caspase-8 with FADD by using immunoprecipitation. As shown in Figure 4B, we detected an increase in the association of FADD with Fas, DR3, or caspase-8 in AKF-D52-treated cells, thereby indicating that AKF-D52 induces DISC formation and initiates the extrinsic pathway. Given that the expression of death receptors or death receptor ligands is regulated by transcription factors, we also examined the activation of nuclear factor kappa-light-chain-enhancer of activated B cells (NF-κB) that was reported as a transcriptional activator of pro-apoptotic genes, such as Fas and FasL [33]. As shown in Figure 4C, Western blot analysis revealed that AKF-D52 elevated the expression of nuclear p65 and the phosphorylation of nuclear p65 and IκBα and reduced the expression of IκBα, indicating that AKF-D52 activated the NF-κB pathway, which regulated Fas and FasL expression in A549 and NCI-H358 cells. These observations thus indicate that AKF-D52-induced DISC formation could be mediated via NF-κB activation in NSCLC cells.

### 3.5. Inhibition of Cytoprotective Autophagy Enhances AKF-D52-Induced Apoptosis in A549 and NCI-H358 Cells

**Figure 5:**
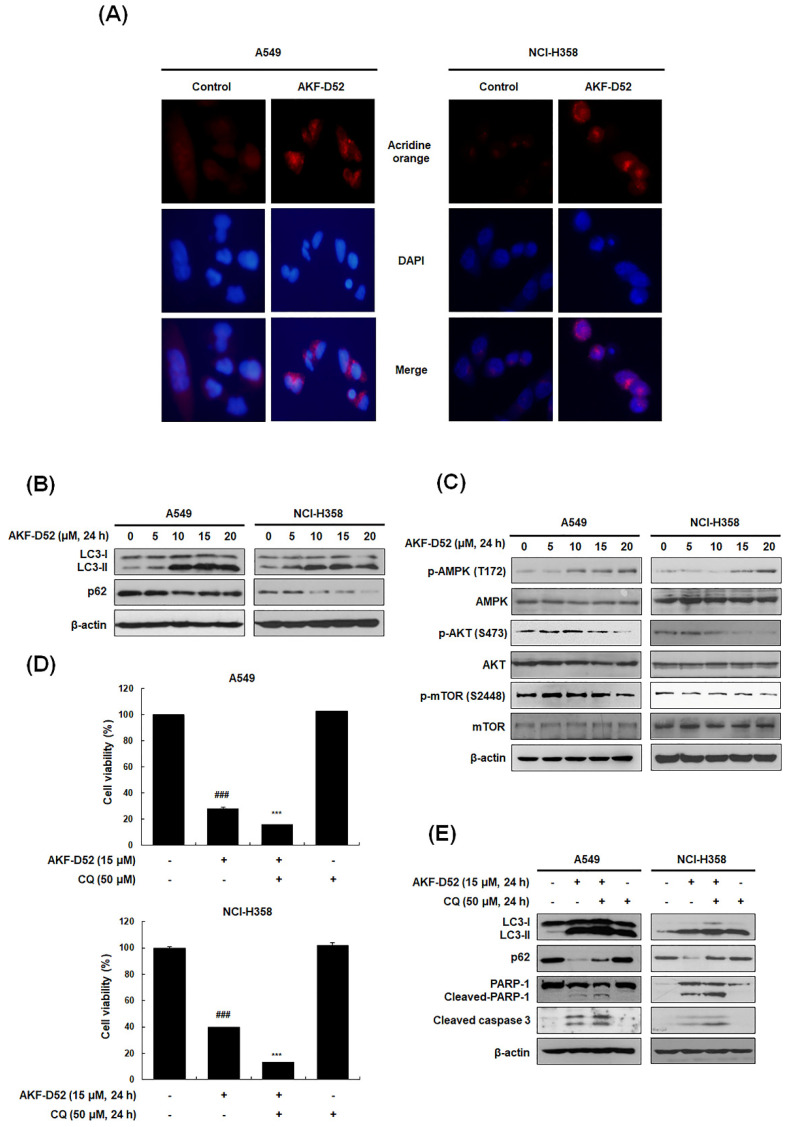
The effects of AKF-D52-induced cytoprotective autophagy on apoptosis in NSCLC cells. A549 and NCI-H358 cells were treated with AKF-D52 (15 μM) for 24 h. (**A**) AKF-D52-treated cells were co-stained with acridine orange and DAPI. Autophagy vacuoles and nuclei were detected by a microscope. (**B**,**C**) Cell lysates were prepared, and Western blot analysis was performed, as described in the *Materials and Methods* section. (**D**) After pretreatment with CQ for 1 h, cells were treated with AKF-D52 (15 μM) for 24 h. Cell viability was measured using the MTT assay. (**E**) Cells were lysed, and Western blot analysis was performed, as described in the *Materials and Methods* section. β-actin was used as an internal control. Data are presented as the mean ± SD of three independent experiments. *^###^ p* < 0.001 vs. the untreated control group, *** p < 0.001 vs. the AKF-D52-treated group.

Autophagy is a self-degradative pathway associated with cell death or survival. In this regard, the conversion of LC3-I to LC3-II is used as a marker for complete autophagosome formation [34]. During autophagic flux activation, the ubiquitin-binding protein p62 is involved in lysosome- or proteasome-dependent protein degradation. To investigate whether AKF-D52 induces autophagy in NSCLC cells, we thus examined the formation of acidic vesicular organelles (AVOs) based acridine orange staining. As shown in Figure 5A, AKF-D52 effectively increased the formation of cytoplasmic AVOs in A549 and H358 cells. In addition, treatment with AKF-D52 resulted in the upregulation of LC3-II and downregulation of p62, indicating the induction of autophagy (Figure 5B). mTOR is a major negative regulator of autophagy, whereby AKT or AMPK induces autophagy via mTOR phosphorylation. Consequently, we investigated the changes in phosphorylated AMPK/AKT/mTOR following AKF-D52 treatment. AKF-D52 was accordingly found to induce AMPK phosphorylation in a dose-dependent manner. Moreover, AKF-D52 treatment had the effect of reducing mTOR and AKT phosphorylation, thereby resulting in the induction of autophagic flux (Figure 5C). These findings thus revealed that AKF-D52 induces autophagy via AMPK/AKT/mTOR signaling pathways. We proceeded to investigate whether AKF-D52-induced autophagy contributes to cell death or survival. As shown in Figure 5D, pretreatment with CQ, an autophagy inhibitor that blocks lysosomal acidification, enhanced AKF-D52-mediated cell death in both assessed cell lines, thus indicating that AKF-D52-induced autophagy plays a cytoprotective role. Pretreatment with CQ also attenuated the AKF-D52-induced reduction in p62 and enhanced the increased expression of LC3-II, cleaved PARP-1, and cleaved caspase-3 (Figure 5E). Collectively, these findings indicate that AKF-D52-induced autophagy plays a cytoprotective role in NSCLC cells.

### 3.6. ROS Plays an Important Role in AKF-D52-Induced Apoptosis in A549 and NCI-H358 Cells

**Figure 6:**
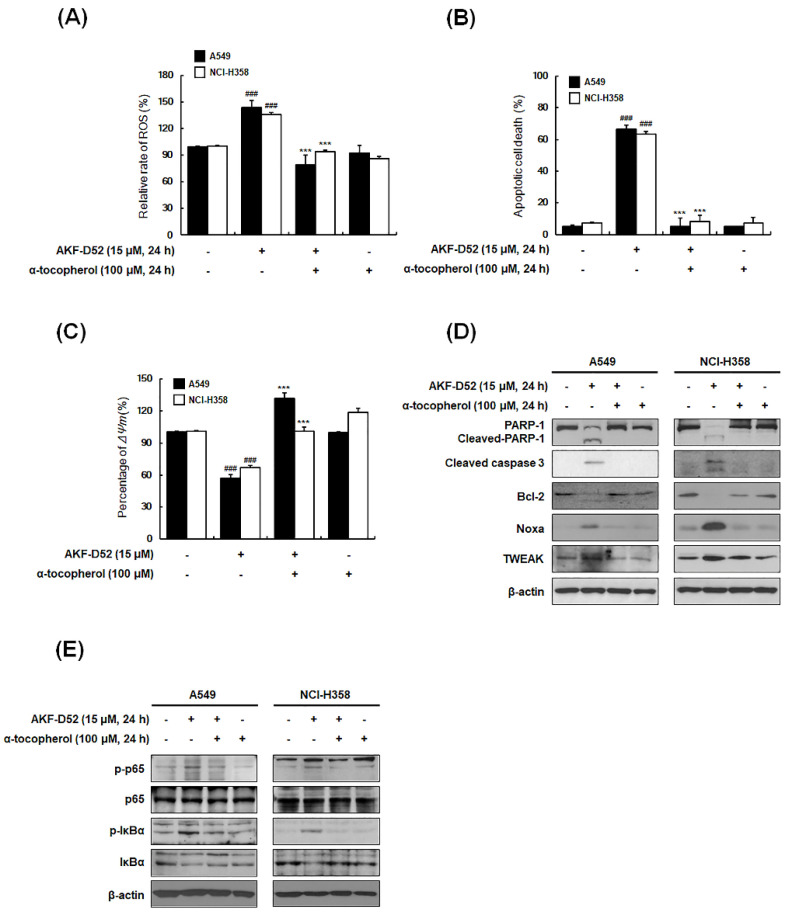
The effects of AKF-D52 on activation of ROS signaling pathways in NSCLC cells. (**A**) Cells were pre-treated with α-tocopherol (an ROS scavenger) for 1 h and then treated with AKF-D52 for 2 h. Cells were stained with H_2_DCFDA, and ROS production was determined by flow cytometry. (**B**) Cells were pre-treated with α-tocopherol and treated with AKF-D52 (15 μM) for 24 h. The externalization of phosphatidylserine in NSCLC cells was determined using an annexin V-FITC/PI assay. (**C**) The disruption of *ΔΨm* was examined by staining with DiOC_6_ and analyzed by flow cytometry. (**D**,**E**) Cells were lysed, and Western blot analysis was performed, as described in the *Materials and Methods* section. β-actin was used as an internal control. Data are presented as the means ± SD of three independent experiments, and significant differences are denoted as *^###^ p* < 0.001 vs. the untreated group, *** p < 0.001 vs. the AKF-D52-treated group.

Given that ROS are important regulators of the autophagy/apoptosis balance in cancer cells [35], we used H_2_DCFDA staining to examine intracellular ROS production in AKF-D52-treated A549 and NCI-H358 cells. AKF-D52 was found to induce ROS generation within 2 h post-treatment, which was abolished by the free radical scavenger α-tocopherol (Figure 6A). Moreover, pretreatment with α-tocopherol completely inhibited AKF-D52-induced apoptosis (Figure 6B) and recovered AKF-D52-induced *ΔΨm* disruption and changes in the levels of protein (cleaved PARP-1, cleaved caspase-3, Bcl-2, Noxa, and TWEAK) expression (Figure 6C,D). Furthermore, in accordance with our hypothesis that AKF-D52 modulates the NF-κB signaling pathway to induce apoptosis and autophagy by regulating intracellular ROS levels, we observed that AKF-D52-induced NF-κB activation was attenuated by α-tocopherol (Figure 6E). Taken together, these findings indicate that AKF-D52-induced apoptosis and NF-κB activation could be mediated by ROS production in NSCLC cells.

### 3.7. AKF-D52 Inhibits Tumor Growth in an A549 Xenograft Mouse Model

**Figure 7:**
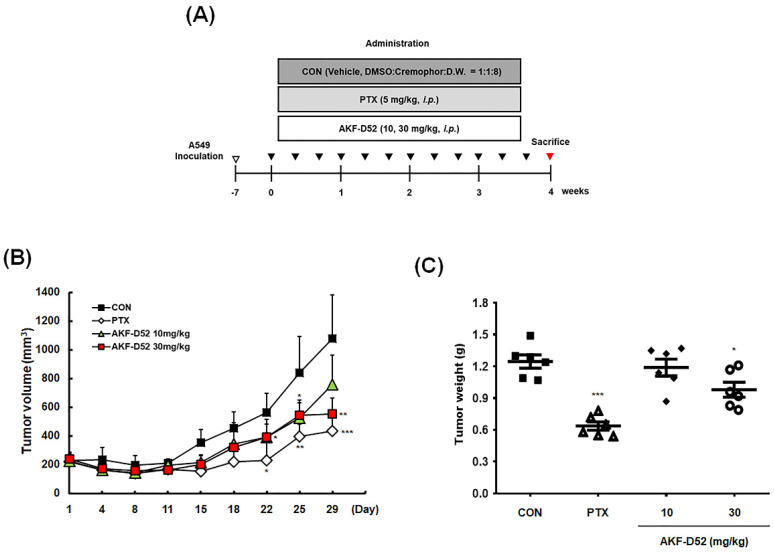

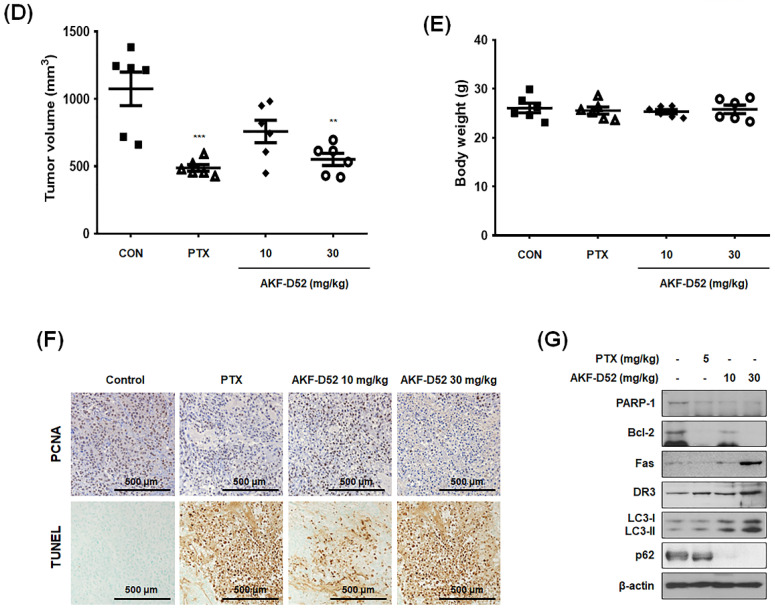
Antitumor activity of AKF-D52 in an A549 xenograft mouse model. (**A**) The scheme of the in vivo experiment. (**B**) Tumor volumes (mm^3^) were measured twice a week during the experimental period. (**C**,**D**) After sacrifice, tumors were separated from the mice, and tumor weight or volume were measured. (**E**) Body weights were measured prior to sacrifice. (**F**) The expression of PCNA was evaluated by IHC analysis, and the induction of apoptosis was examined based on a TUNEL assay using tumor sections. (**G**) Isolated tumor tissues were homogenized, and Western blot analysis was performed, as described in the *Materials and Methods* section. β-actin was used as an internal control. Data are presented as the means ± SD of values from measurement (*n* = 6), and significant differences are denoted as * p < 0.05, ** p < 0.01, *** p < 0.001 vs. the vehicle-treated group.

Having investigated the inhibitory effects of AKF-D52 in vitro, we went on to examine the antitumor effects of AKF-D52 in A549 cell-implanted BALB/c nude mice. A549 cell-bearing mice were treated with vehicle, paclitaxel (5 mg/kg, i.p., three times per week), or AKF-D52 (10 or 30 mg/kg, i.p., three times per week) for 4 weeks (Figure 7A). Compared with the increased tumor volume in the vehicle-treated group, we observed a significant reduction in tumor volume after day 22 in both paclitaxel- and AKF-D52 (30 mg/kg)-treated groups (Figure 7B). Data obtained for the weight and volume of isolated tumor tissues revealed significant reductions in the average tumor weight in 30 mg/kg AKF-D52- (0.98 ± 0.17 g, *p-*value: 0.0187) and paclitaxel (0.64 ± 0.10 g, *p-*value < 0.0001)-treated groups, compared with the vehicle-treated group (1.25 ± 0.15 g) (Figure 7C). Similarly, compared with the vehicle-treated group (1075.9 ± 305.1 mm^3^), there were reductions in tumor volume in the AKF-D52- (10 mg/kg: 759.1 ± 204.4 mm^3^; 30 mg/kg: 551.8 ± 110.4 mm^3^, *p-*value: 0.0027) and paclitaxel (435.0 ± 36.7 mm^3^, *p-*value: 0.0009)-treated group (Figure 7D). Consistent with the cell-based results, AKF-D52 was found to modulate the expression of proteins related to apoptosis and autophagy induction in tumor tissues. As shown in Figure 7F, AKF-D52-treated tumor tissues showed reduced expression of proliferating cell nuclear antigen (PCNA), a marker protein for cell proliferation. Furthermore, the results of a TUNEL assay and Western blotting, showing PARP-1 cleavage, Bcl-2 downregulation, and Fas and DR3 upregulation, revealed that AKF-D52 induces apoptosis in lung tumor tissues. In addition, we found that AKF-D52 treatment had a regulatory effect on autophagy markers (LC3 and p62) in tumor tissues of xenograft model mice (Figure 7G). To assess potential AKF-D52 toxicity, we monitored body weight (Figure 7E) and serum levels of AST, ALT, and BUN in the A549 cell-bearing mice (Supplementary Figure S1A), and accordingly, we established that the effective doses of AKF-D52 (10 or 30 mg/kg, i.p.) did not significantly affect the body weight or serum levels of AST, ALT, or BUN. These findings would therefore appear to indicate the absence of AKF-D52 systemic toxicity in vivo.

## 4. Discussion

Programmed cell death (PCD) is a cell-active self-destruction mechanism that can be further classified into three major types, namely apoptosis, necrosis, and autophagy [36,37]. Among the PCD types, apoptosis removes impaired cells in an orderly and efficient manner, resulting in nuclear condensation, fragmentation, and dynamic membrane blebbing [38]. Given that apoptosis inhibits cancer cell growth with minimal effects on normal cells, compounds that induce apoptosis might thus have potential utility in cancer treatment [39]. In a previous study, we found that AKF-D52 showed growth-inhibitory and cytotoxic effects on multiple cancer cell lines, including lung, colorectal, skin, ovarian, renal, prostate, breast, and hematologic cancers [26]. In the present study, we investigated the mechanisms underlying the antiproliferative effects of AKF-D52 at the molecular level, both in vitro and in vivo. The results obtained revealed that the cytotoxicity of AKF-D52 in A549 (Figure S1) and NCI-H358 cells (IC_50_: 4.49 μM and 6.62 µM, respectively) was more selective for NSCLC cells than for normal lung epithelial cells (BEAS-2B), and this cytotoxicity was attributable to the induction of apoptosis characterized by DNA fragmentation and externalization of phosphatidylserine.

Caspases are endoproteases that contribute to the regulation of cell death and inflammation. In general, activation of the caspase cascade requires initiator caspases, such as caspase-8, -9, and -10, and effector caspases, such as caspase-3, -6, and -7, the latter of which cleave several vital substrates, such as PARP and laminA/C, which leads to apoptosis [40].

In contrast, cellular responses related to mitochondrial outer membrane permeabilization (MOMP) lead to the release of Endo G and AIF. These mitochondrial proteins translocate to the nucleus and cause large-scale DNA fragmentation, resulting in caspase-independent apoptosis [30,41]. In agreement with these findings, AKF-D52 was found not only to activate caspase-8 and -9 and the downstream caspase-3 and lead to the cleavage of PARP but also to induce the translocation of AIF/Endo G, indicating that both caspase-dependent and -independent pathways are involved in the AKF-D52-induced apoptosis. Based on these observations, we speculated that AKF-D52-induced apoptosis might be mediated via both the mitochondrial and death receptor pathways.

Mitochondria play pivotal roles in the apoptotic pathway, including the maintenance of Bcl-2 family protein balance, caspase activation, and induction of chromosomal fragmentation [42]. The mitochondrial cytochrome *c* released into the cytosol interacts with apoptotic peptidase activating factor 1 (APAF-1) to form an apoptosome that activates caspase-9 and -3 to induce apoptosis [43]. In our study, we found that AKF-D52-regulated expression of Bcl-2 family proteins (Bid, Bim, Noxa, Bad, Bcl-2) leads to *ΔΨm* disruption and the release of mitochondria-related proteins, including cytochrome *c* and Smac/Diablo, which consequently induces the intrinsic apoptosis pathway. These data indicate that AKF-induced caspase-dependent or caspase-independent apoptosis is caused by an increase in the levels of BH3-only proteins (Bad, Bid, Bim, and Noxa) and *ΔΨm* disruption.

Apoptosis is also alternatively activated via an extrinsic pathway involving death receptors, which bind to their corresponding death ligands such as Fas L (which binds to Fas) or TWEAK (which binds to DR3). The binding of FasL to Fas or TNF-ligand/TNF receptor binding induces the recruitment of TNF receptor type 1-associated death domain protein, FADD, and receptor-interacting protein [6], thereby generating the DISC, leading to the initiation of apoptosis via caspase-8 activation [44]. We found that AKF-D52 treatment promotes the upregulated expression of DISC component proteins (Fas, FasL, TWEAK, DR3, and FADD) and elevates the levels of DISC binding.

FasL expression is transcriptionally regulated by a range of transcriptional factors, including interferon regulatory factor-1, nuclear factor in activated T-cells, and NF-κB [45]. Several studies on the pro-apoptotic roles of NF-κB [46,47] have shown that NF-κB activation results in the elevated expression of Fas, FasL [48,49], or the TNF-related apoptosis-inducing ligand, and eventually DISC formation and apoptosis [50]. In the present study, we established that AKF-D52 triggers NF-κB activation via the degradation of IκB and nuclear translocation of p-p65 NF-κB subunit in A549 and NCI-H358 cells, thereby indicating that AKF-D52-induced NF-κB activation might be associated with pro-apoptotic roles.

The balance between cell survival and cell death is an important factor contributing to the maintenance of cellular homeostasis. Notably, autophagy contributes to the control of cell homeostasis, inducing either cell survival (cytoprotective autophagy) [51,52] or cell death (autophagic cell death) [53,54]. In the present study, we observed that AKF-D52 treatment triggers autophagy by increasing AVOs’ formation and LC3-II accumulation, coupled with a reduction in the expression of p62 in A549 and NCI-H358 cells. Importantly, the results revealed that CQ, a pharmacological autophagy inhibitor, enhances AKF-D52-induced cell death, as well as caspase-3 and PARP cleavage, thus indicating that AKF-D52-induced cytoprotective autophagy impedes AKF-D52-induced apoptosis in A549 and NCI-H358 cells. Consistent with the findings of previous investigations using small interfering RNA or pharmacological agents [55,56,57,58], we found that non-toxic doses of autophagy inhibitor could be potentiated by co-treatment with AKF-D52, which show synergism in A549 and NCI-H358 lung cancer cells. Accordingly, the effects of the combined application of AKF-D52 and autophagy inhibitors in a range of cancer or drug-resistant cell lines warrant further investigation.

Intracellular ROS play a prominent role in carcinogenesis and cancer development [59]. However, when exceeding certain levels, ROS evoke apoptotic responses mediated via several signaling pathways involving MAPK, STAT3, FOXO, or NF-κB [35,60,61,62]. A number of studies have shown that the excess production of ROS suppresses the proliferation of lung cancer cells and induces apoptosis and autophagy [63,64,65]. ROS production in close proximity to lipid membranes causes lipid peroxidation, which has been implied as a critical event during ROS-mediated cancer cell death [66,67]. Protection against lipid peroxidation is provided by α-tocopherol, which has been described to act as a non-enzymatic hydrophobic antioxidant and possesses protective effects against cell death [68,69]. Consistent with these results, we found that α-tocopherol suppressed AKF-D52-induced ROS production and apoptosis and that treatment with NAC was ineffective in reducing these preventive effects, indicating that α-tocopherol probably contributed to preventing AKF-D52-induced lipid peroxidation. Therefore, further investigation is required to understand the exact mechanism of how α-tocopherol inhibits AKF-D52-induced apoptosis through scavenging membrane peroxyl radicals in human NSCLC cells. These findings are consistent with those reported in a recent study showing crosstalk between ROS and NF-κB signaling [70]. α-Tocopherol is a potent chain-breaking antioxidant that resides in cellular membranes, wherein it maintains membrane integrity by inhibiting lipid peroxidation [71]. We found that α-tocopherol suppressed AKF-D52-induced ROS production and apoptosis and that treatment with NAC was ineffective in reducing these preventive effects, indicating that α-tocopherol probably contributed to preventing AKF-D52-induced lipid peroxidation. Consequently, it would be of particular interest to investigate whether AKF-D52 affects lipid peroxidation in human NSCLC cells.

In our previous study, AKF-D52 specifically inhibits FMS, also known as CSF-1R, which is known as a class 3 transmembrane tyrosine kinase receptor [72]. Notably, the overexpression of FMS activates the phosphoinositide 3-kinase/Akt pathway, which promotes cancer survival [73] and is related to chemoresistance in lung cancer [74]. Therefore, further investigation is needed for unraveling the relationship between FMS inhibiting activity and the apoptosis-inducing molecular mechanism of AKF-D52.

To verify the in vivo efficacy of AKF-D52, we established a BALB/c nude mouse xenograft model and subsequently observed that intraperitoneal administration of AKF-D52 (10 or 30 mg/kg) significantly reduced tumor volume and weight. Consistent with our in vitro findings, AKF-D52-induced apoptosis was confirmed in tumor tissues of the A549 xenograft model mice based on IHC with PCNA and TUNEL assays. In addition, AKF-D52 was observed to effectively reduce the levels of PARP, Bcl-2, and p62 proteins and upregulate the death receptors (Fas and DR3) and LC3-II, thus indicating that AKF-D52-induced apoptosis and autophagy may be essential mechanisms in inhibiting the progression of non-small cell lung tumors. In addition, by monitoring body weight and the serum levels of AST, ALT, and BUN in model mice, we established that AKF-D52 does not cause any abnormal changes in these parameters, thereby tending to indicate the absence of hepatotoxicity and nephrotoxicity. These findings accordingly highlight the potential applicability of AKF-D52 as an anticancer drug that lacks severe adverse effects.

Even we have investigated the tumor-suppressive effect and underlying molecular mechanism of AKF-D52 in vitro and in vivo, including our previous reports, we did not yet proceed with any clinical trial of this compound. However, our team also aims to apply the IND (Investigational New Drug) following ICH (International Council for Harmonization of Technical Requirements for Pharmaceuticals for Human Use) guidelines in the near future after accumulating preclinical research data, including pharmacokinetic and toxicological studies. This report raised the possibility of the AKF-D52 potency against NSCLC lung cancers.

## 5. Conclusions

In summary, in this study, we demonstrated that AKF-D52 exerts its anticancer effects via intracellular ROS production and NF-κB activation, resulting in apoptotic cell death via intrinsic and extrinsic apoptotic pathways and cytoprotective autophagy in NSCLC cells. Collectively, our findings regarding the efficacy and molecular mechanisms of AKF-D52 indicate that this compound is a potentially promising pharmacological tool for the treatment of NSCLC.

## Data Availability

The data are available on request from the corresponding author.

## Supplementary Materials

The following are available online at https://www.mdpi.com/article/10.3390/cancers13225849/s1, Figure S1: Evaluation of the toxicity of AKF-D52 and paclitaxel in an A549 xenograft model, Figure S2: Original images and densitometer analysis of Western blot data in Figure 2A, Figure S3: Original images and densitometer analysis of Western blot data in Figure 2C, Figure S4: Original images and densitometer analysis of Western blot data in Figure 3A, Figure S5: Original images and densitometer analysis of Western blot data in Figure 3C, Figure S6: Original images and densitometer analysis of Western blot data in Figure 4A, Figure S7: Original images and densitometer analysis of Western blot data in Figure 4B, Figure S8: Original images and densitometer analysis of Western blot data in Figure 4C, Figure S9: Original images and densitometer analysis of Western blot data in Figure 5B, Figure S10: Original images and densitometer analysis of Western blot data in Figure 5C, Figure S11: Original images and densitometer analysis of Western blot data in Figure 5E, Figure S12: Original images and densitometer analysis of Western blot data in Figure 6D, Figure S13: Original images and densitometer analysis of Western blot data in Figure 6E, Figure S14: Original images and densitometer analysis of Western blot data in Figure 7G.
