# Supplementary material for: AKF-D52, a Synthetic Phenoxypyrimidine-Urea Derivative, Triggers Extrinsic/Intrinsic Apoptosis and Cytoprotective Autophagy in Human Non-Small Cell Lung Cancer Cells
Source: Cancers (Basel). 2021 Nov 22;13(22):5849. doi: 10.3390/cancers13225849 (PMC8616202; doi:10.3390/cancers13225849)
Supplement: Supplementary file 1 [file cancers-13-05849-s001.zip › cancers-1440416-SI.pdf]

Supplementary Figures

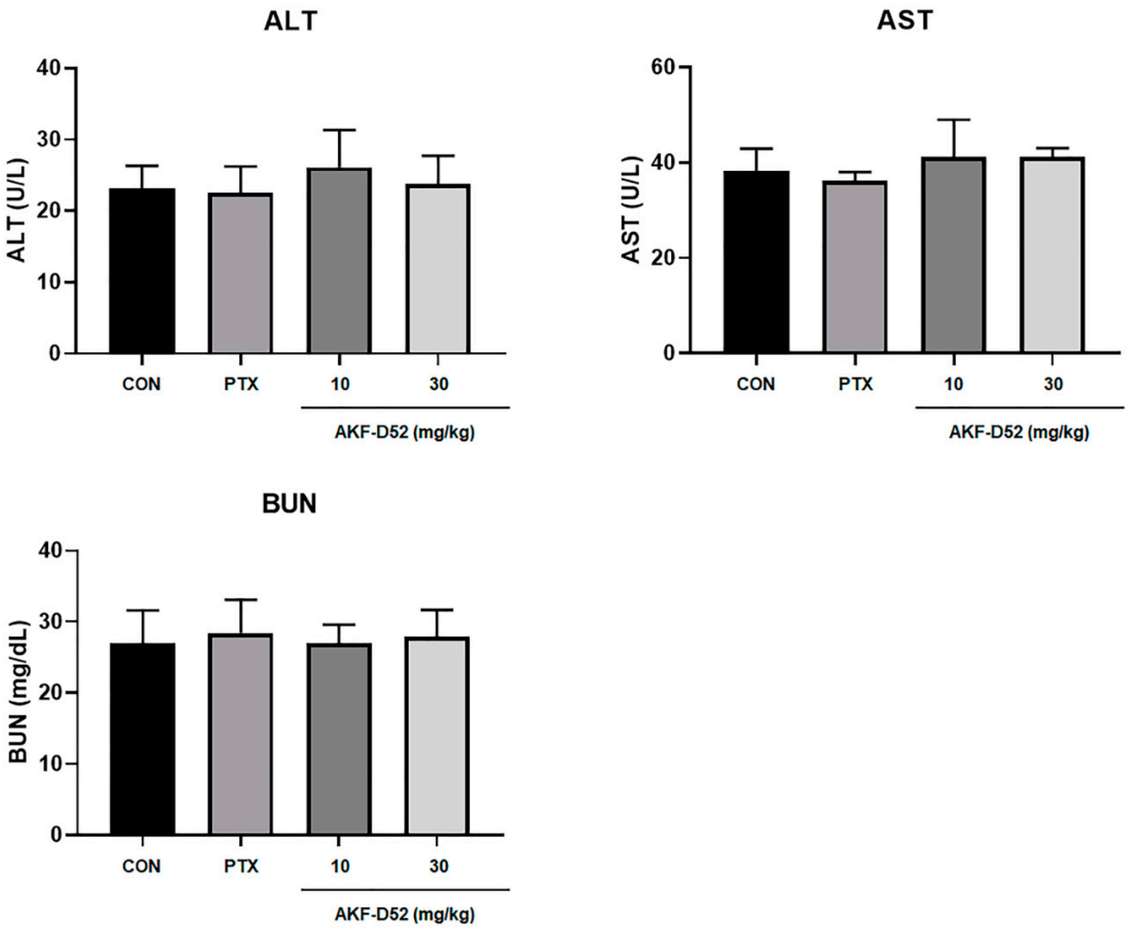

Figure S1. Evaluation of the toxicity of AKF-D52 and paclitaxel in an A549 xenograft model. ALT, AST, and BUN levels were examined using plasma samples from the mice of each group.

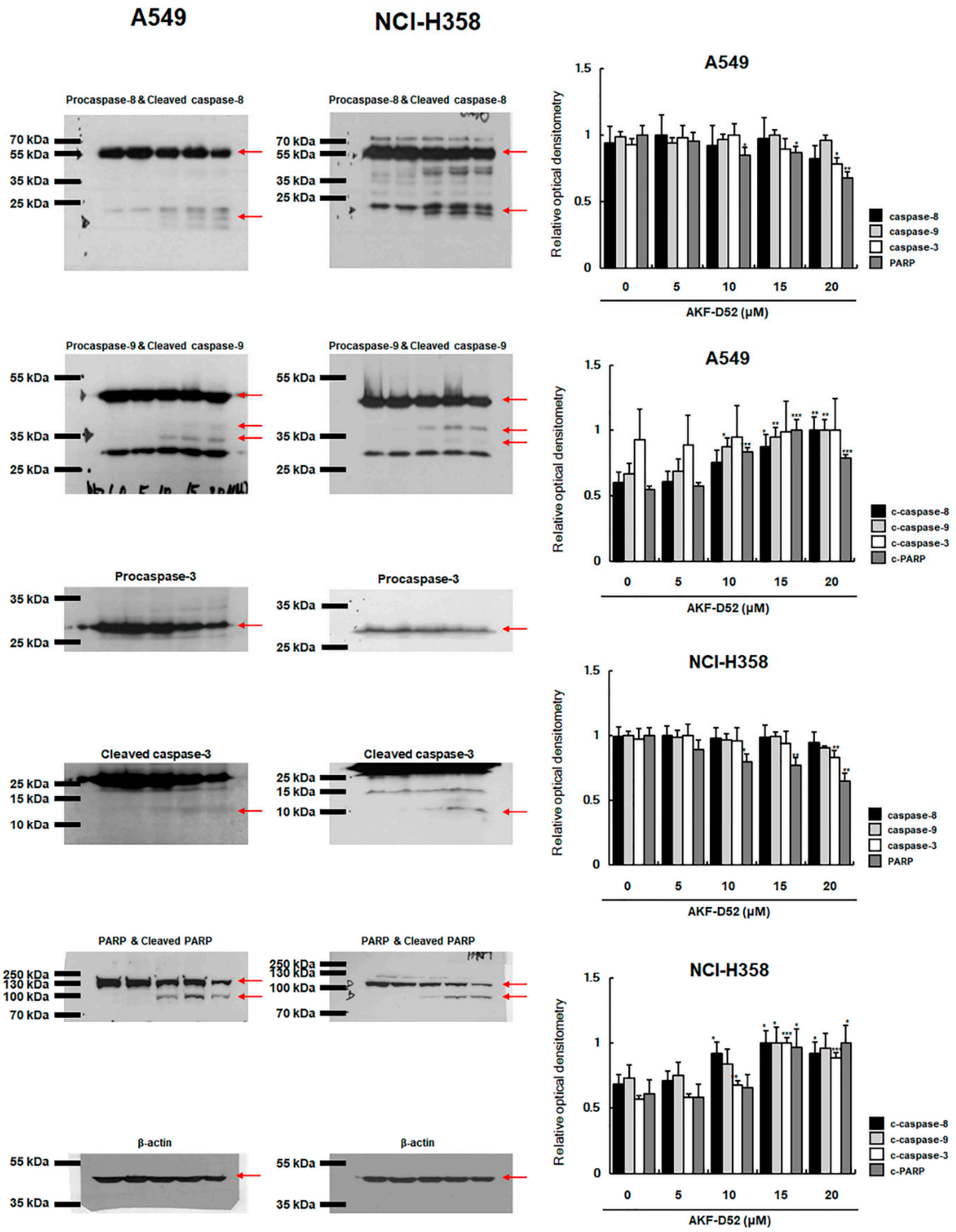

Figure S2. Original images and densitometer analysis of Western blot data in Figure 2A.

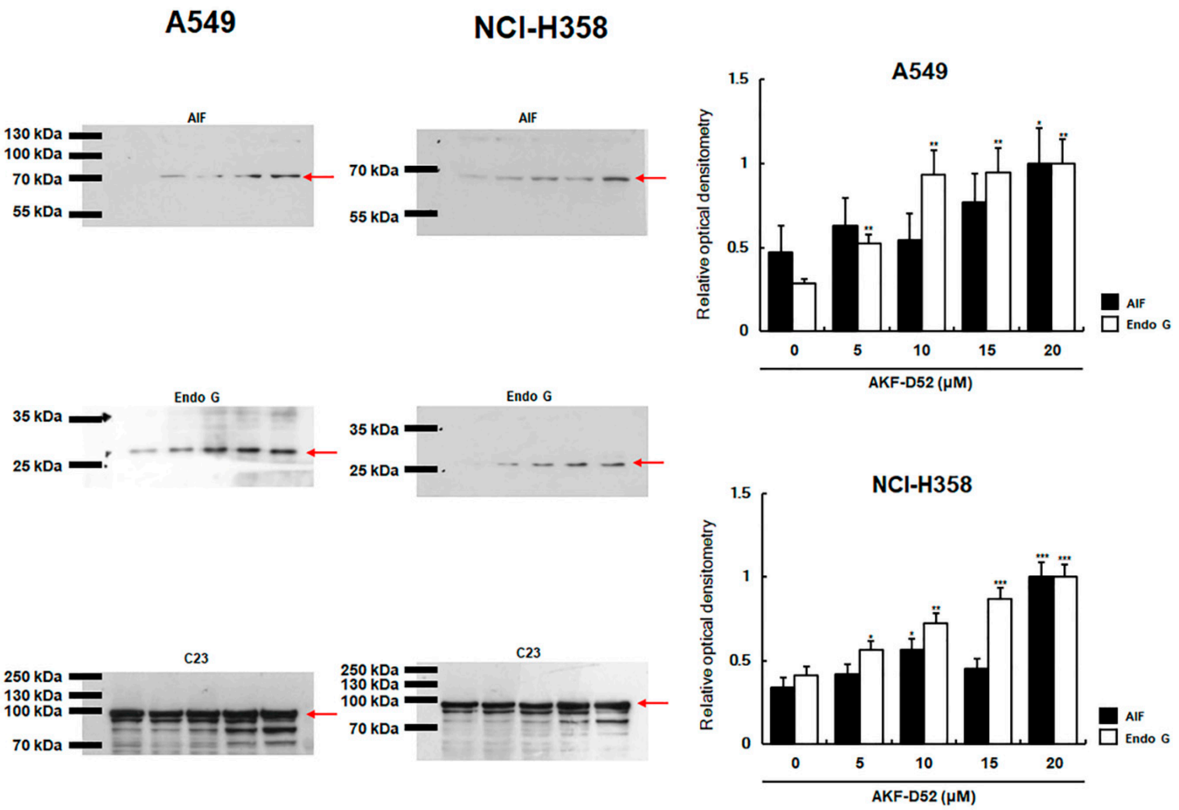

Figure S3. Original images and densitometer analysis of Western blot data in Figure 2C.

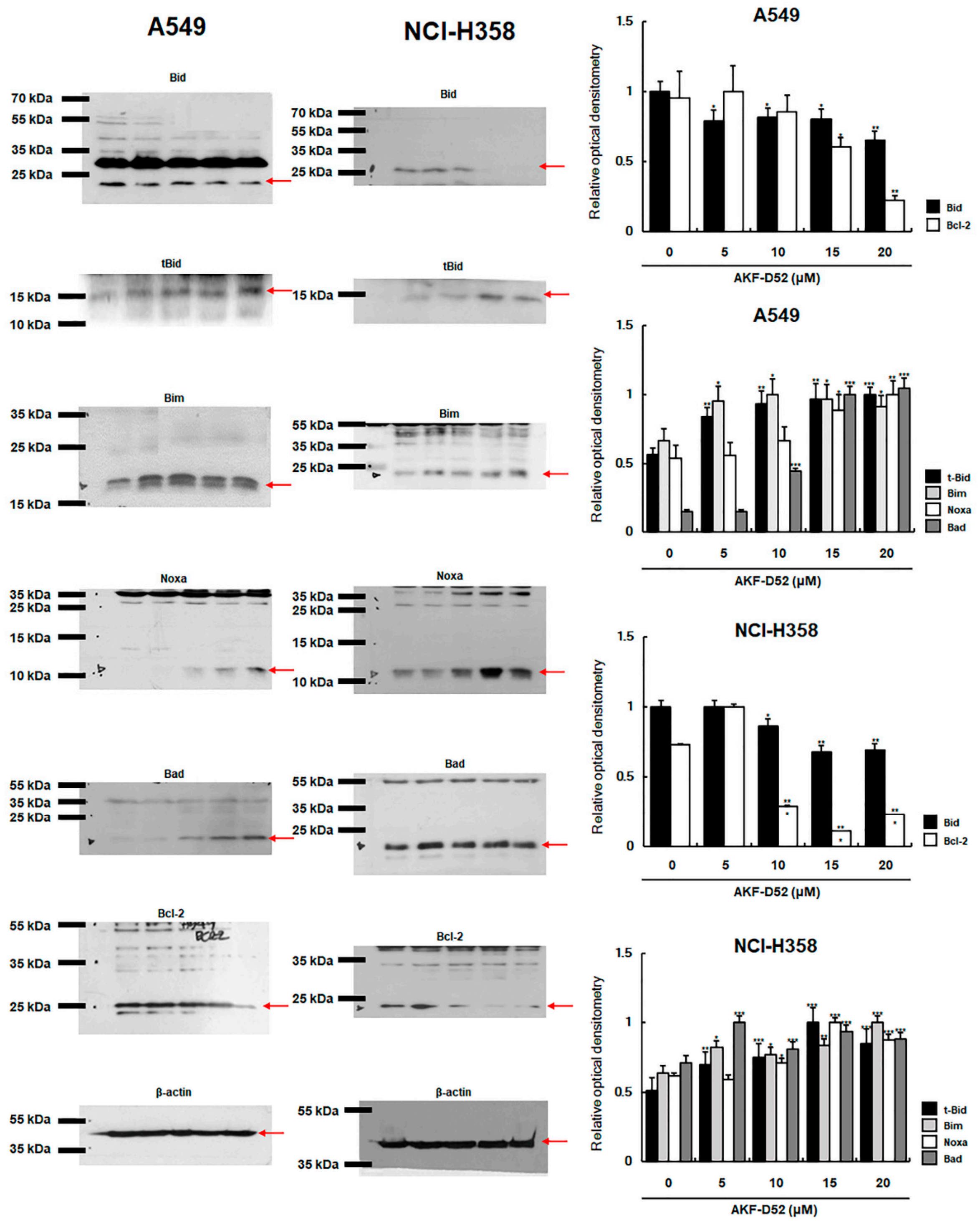

Figure S4. Original images and densitometer analysis of Western blot data in Figure 3A.

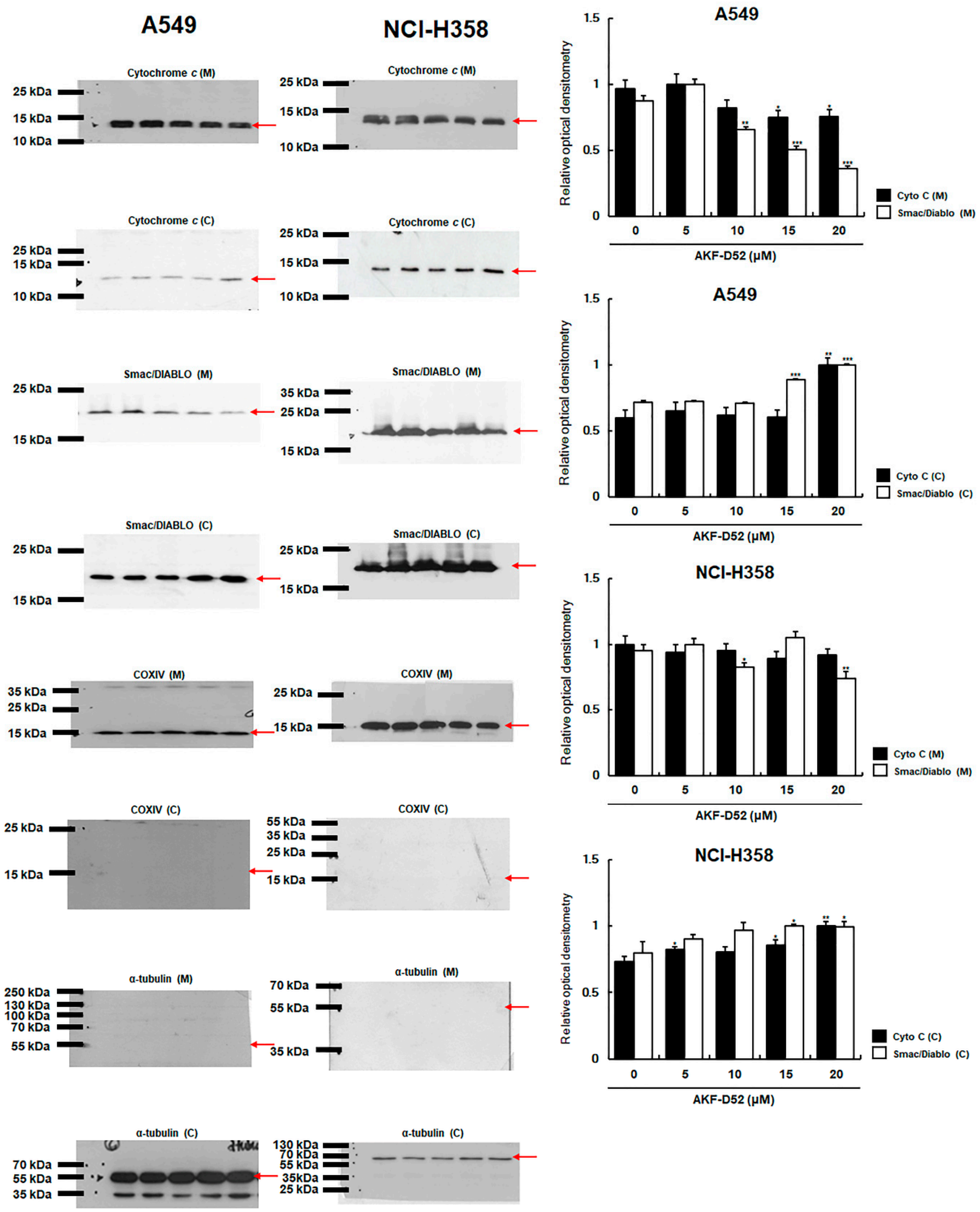

Figure S5. Original images and densitometer analysis of Western blot data in Figure 3C.

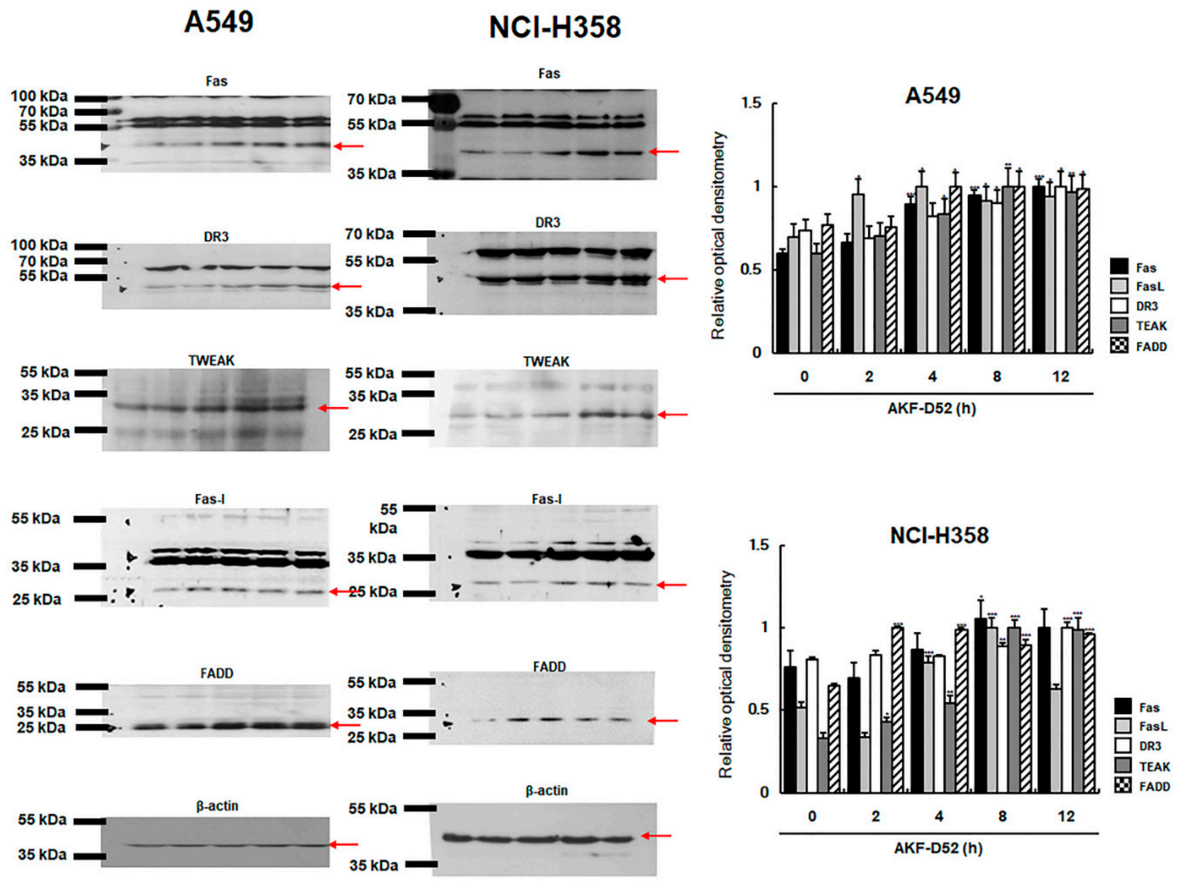

Figure S6. Original images and densitometer analysis of Western blot data in Figure 4A.

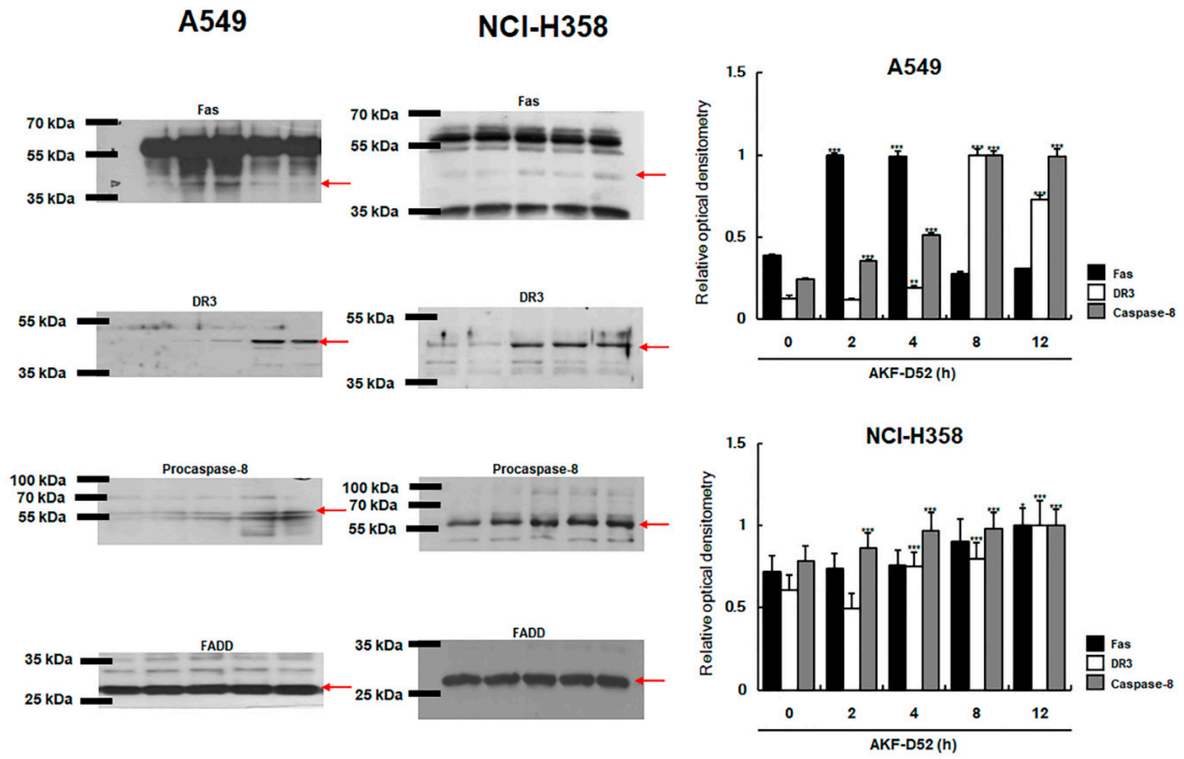

Figure S7. Original images and densitometer analysis of Western blot data in Figure 4B.

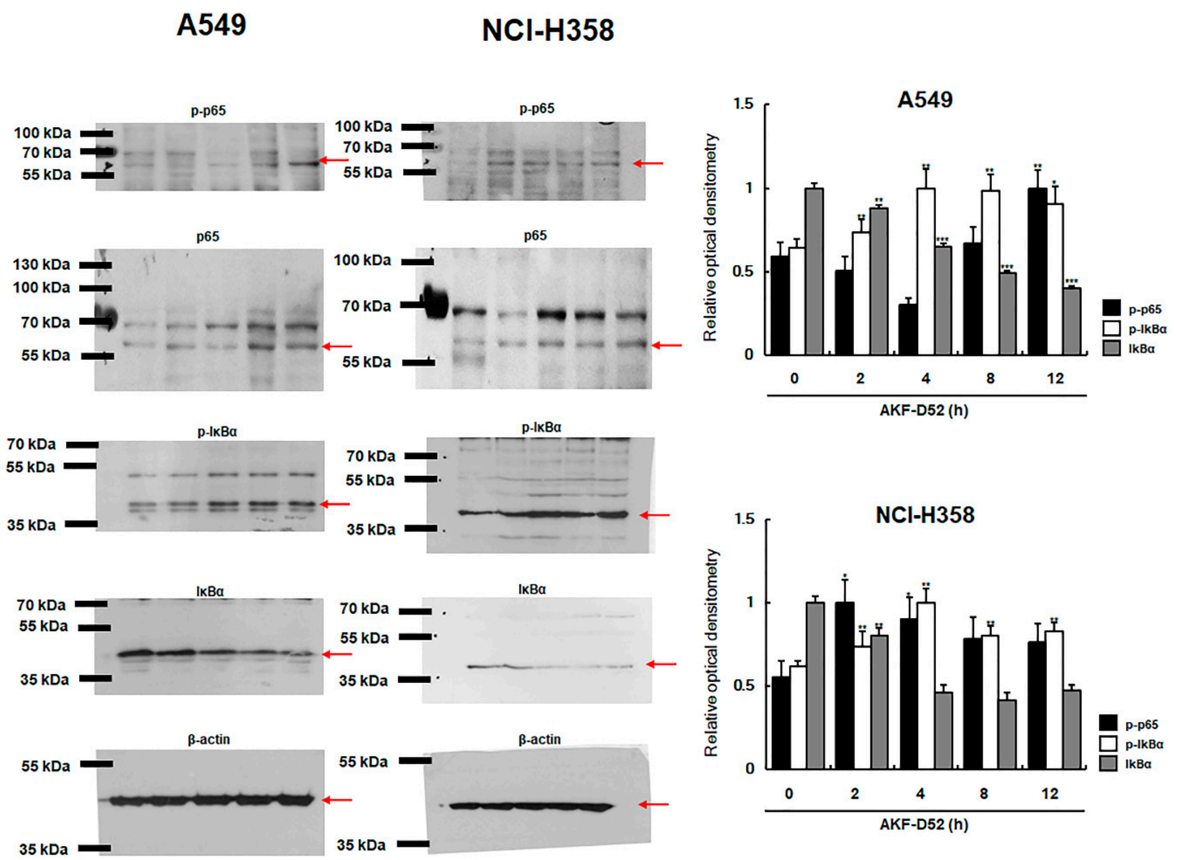

Figure S8. Original images and densitometer analysis of Western blot data in Figure 4C.

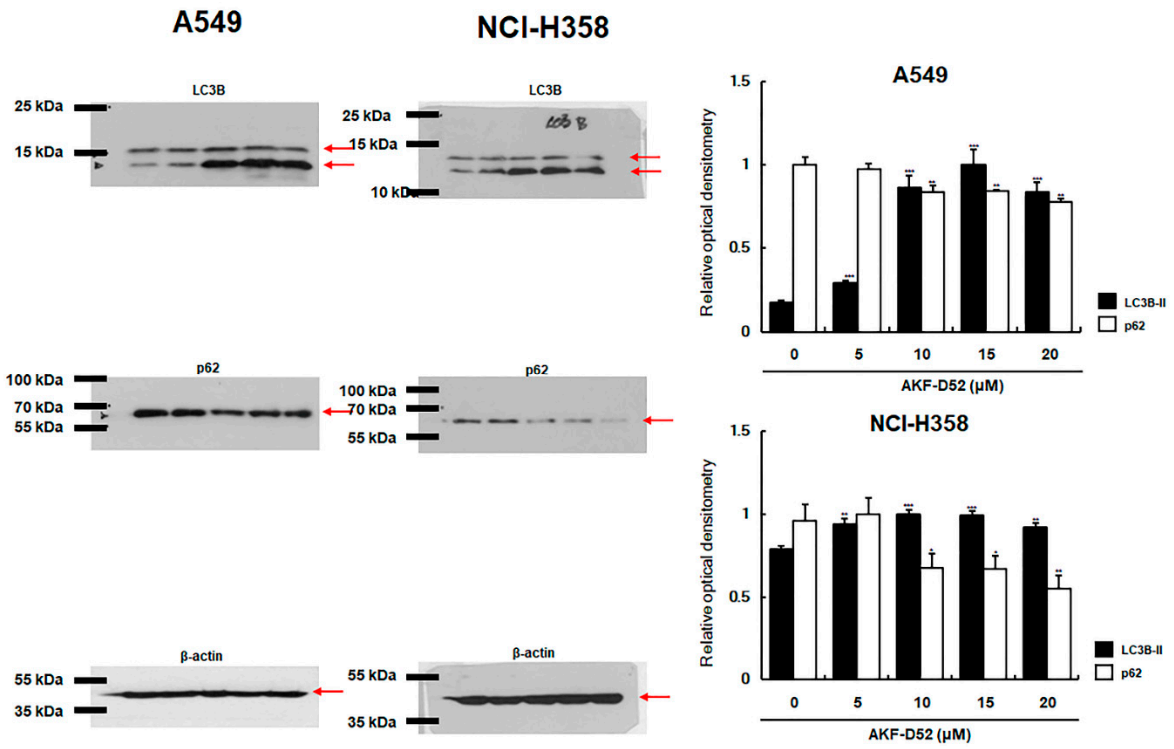

Figure S9. Original images and densitometer analysis of Western blot data in Figure 5B.

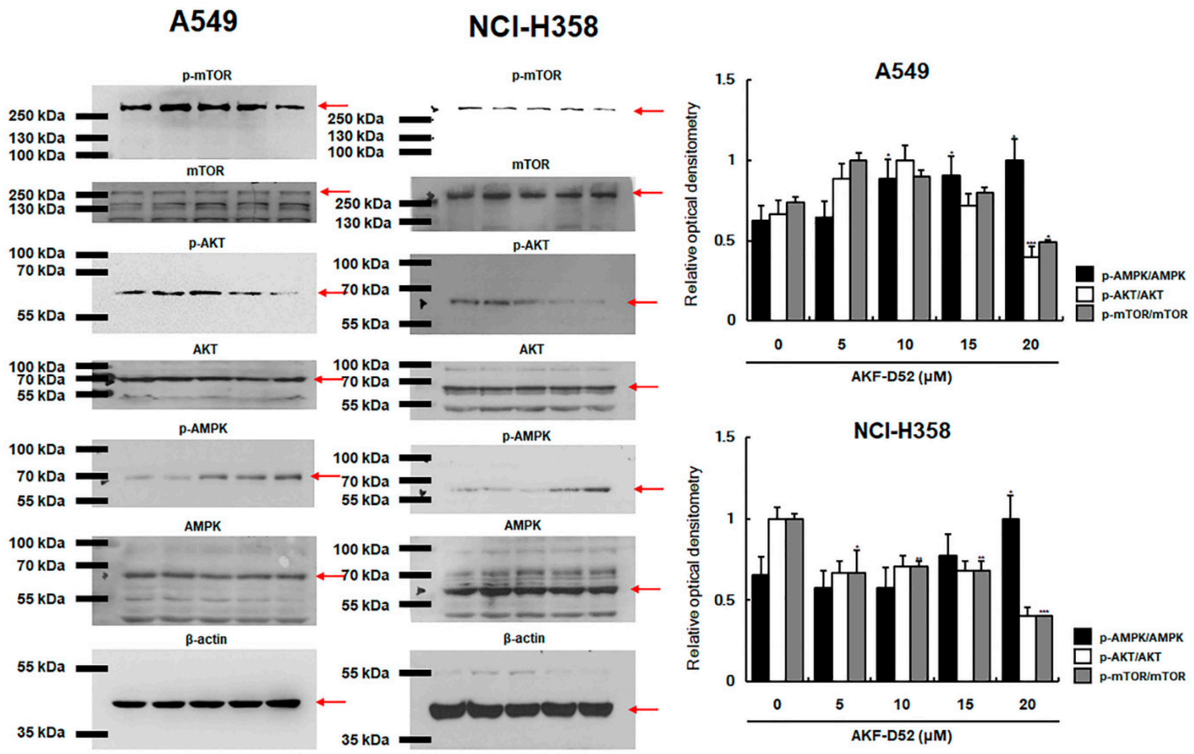

Figure S10. Original images and densitometer analysis of Western blot data in Figure 5C.

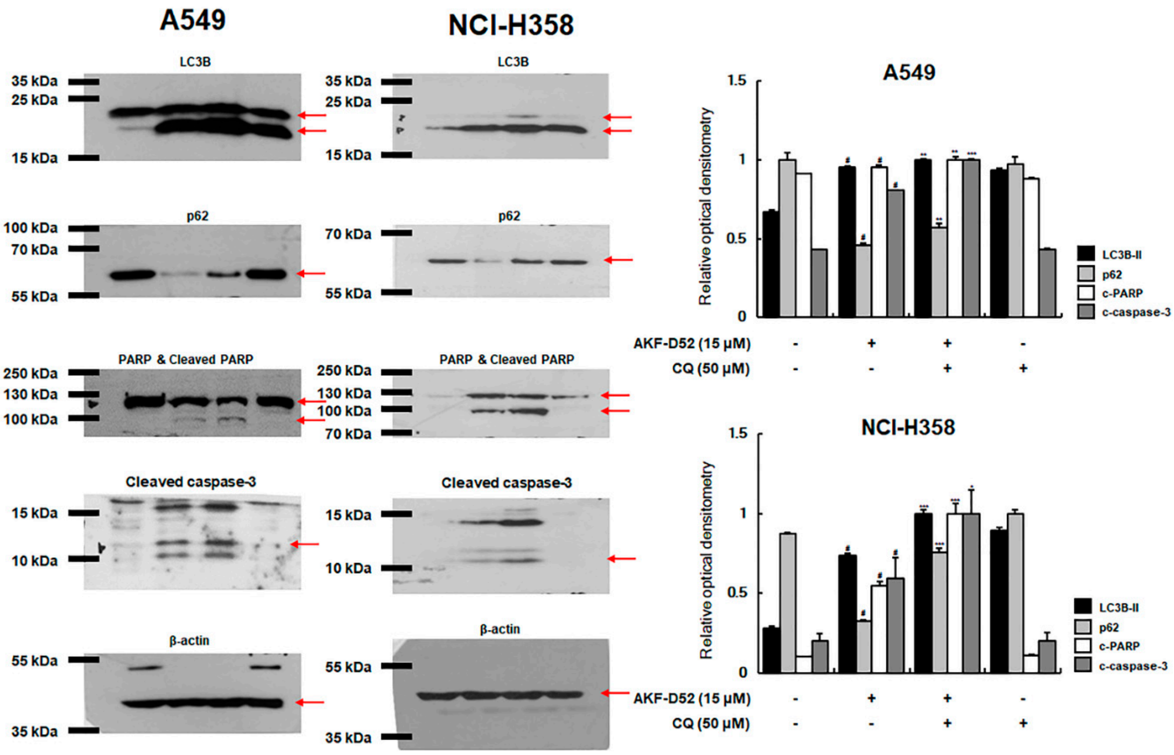

Figure S11. Original images and densitometer analysis of Western blot data in Figure 5E.

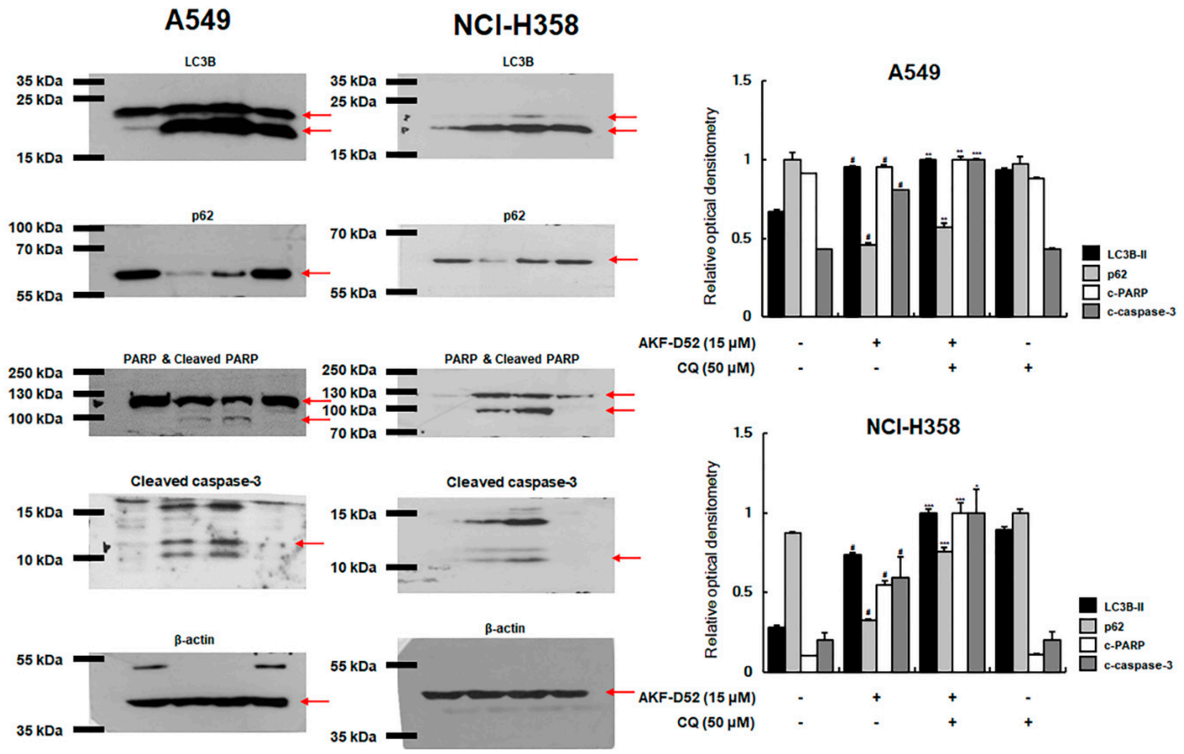

Figure S12. Original images and densitometer analysis of Western blot data in Figure 6D.

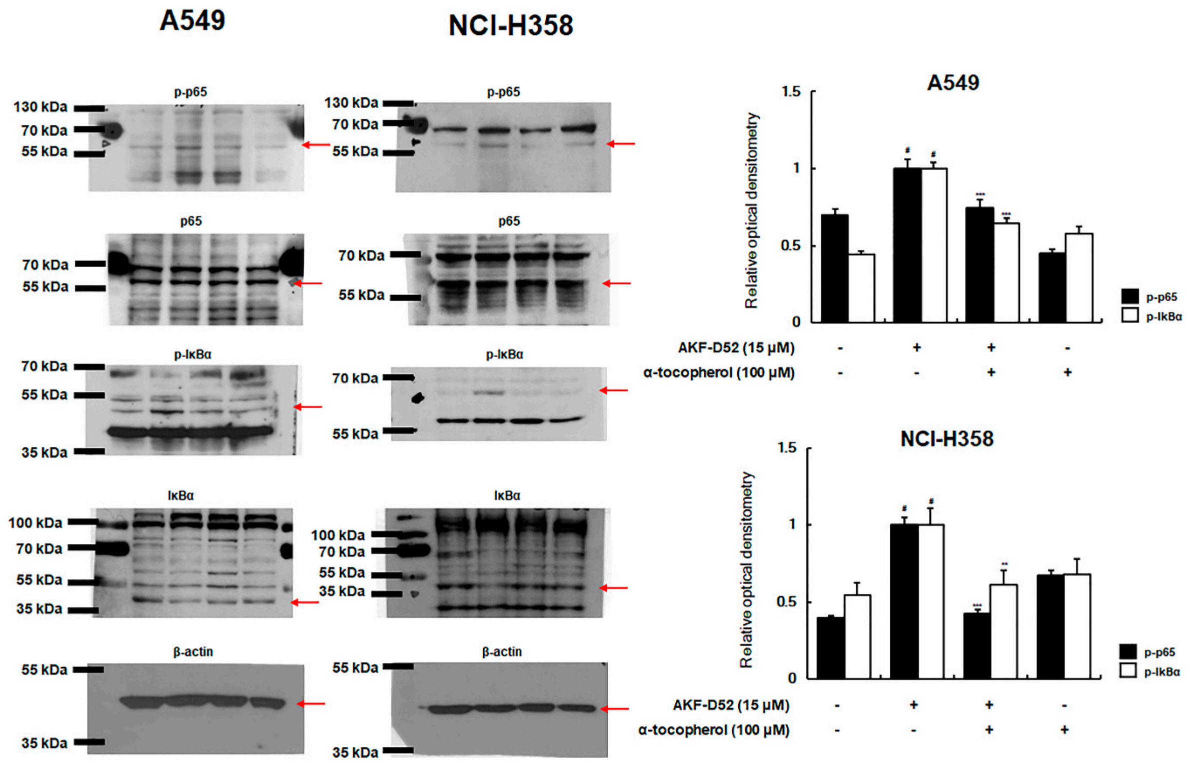

Figure S13. Original images and densitometer analysis of Western blot data in Figure 6E.

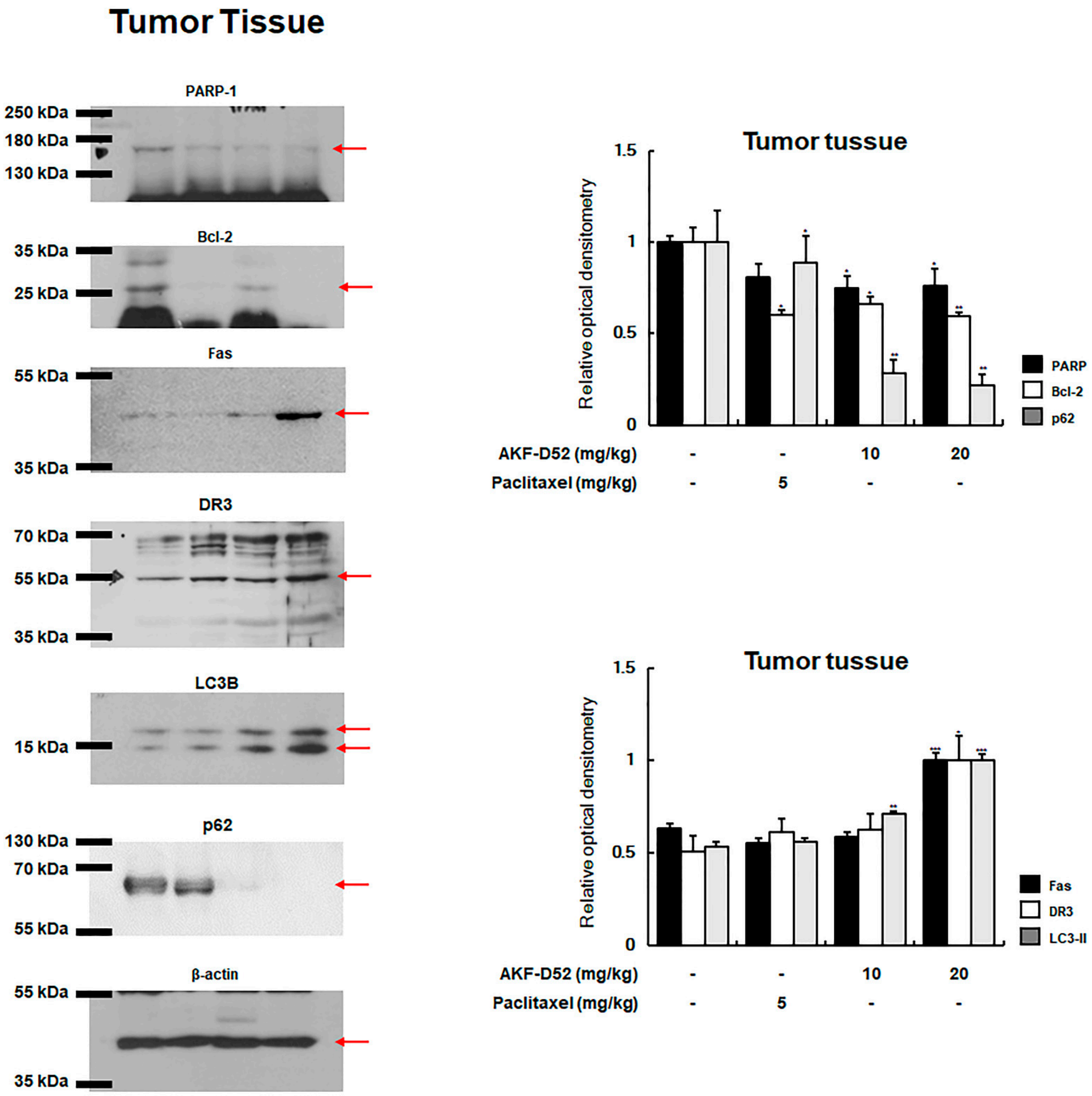

Figure S14. Original images and densitometer analysis of Western blot data in Figure 7G.
